# South America holds the greatest diversity of native daisies (Asteraceae) in the world: an updated catalogue supporting continental-scale conservation

**DOI:** 10.3389/fpls.2024.1393241

**Published:** 2024-05-30

**Authors:** Andrés Moreira-Muñoz, Marcelo Monge, Mariana A. Grossi, Fabio Andrés Ávila, Vanezza Morales-Fierro, Gustavo Heiden, Berni Britto, Stephan Beck, Jimi N. Nakajima, Vanina G. Salgado, Juan Facundo Rodríguez-Cravero, Diego G. Gutiérrez

**Affiliations:** ^1^ Instituto de Geografía, Pontificia Universidad Católica de Valparaíso, Valparaíso, Chile; ^2^ Laboratório de Sistemática Vegetal, Instituto de Biologia, Universidade Federal de Uberlândia, Uberlândia, Brazil; ^3^ Laboratório de Sistemática Vegetal, Instituto de Biologia, Universidade Estadual de Campinas, Campinas, Brazil; ^4^ División Plantas Vasculares, Museo de La Plata, Facultad de Ciencias Naturales y Museo, Universidad Nacional de La Plata, La Plata, Argentina; ^5^ Laboratorio de Morfología Comparada de Espermatófitas, Facultad de Ciencias Agrarias y Forestales, Universidad Nacional de La Plata, La Plata, Argentina; ^6^ New York Botanical Garden, New York, NY, United States; ^7^ The Graduate Center, City University of New York, New York, NY, United States; ^8^ Herbario EIF & Laboratorio de Evolución y Sistemática, Facultad de Ciencias Forestales y de la Conservación de la Naturaleza, Universidad de Chile, Santiago, Chile; ^9^ Museo Nacional de Historia Natural, Santiago, Chile; ^10^ Embrapa Clima Temperado, Pelotas, Brazil; ^11^ Máster Conservación y Gestión del Medio Natural, Universidad de Cádiz, Cádiz, Spain; ^12^ Herbario Nacional de Bolivia, Universidad Mayor de San Andrés, La Paz, Bolivia; ^13^ División Plantas Vasculares, Museo Argentino de Ciencias Naturales, Consejo Nacional de Investigaciones Científicas y Técnicas, Buenos Aires, Argentina

**Keywords:** Compositae (Asteraceae), large-scale conservation, diversity, IUCN, Brazilian Plateau, Andes, megadiverse countries

## Abstract

Asteraceae is the world’s richest plant family and is found on all continents, in environments ranging from the coast to the highest mountains. The family shows all growth forms and, as in other angiosperm families, species richness is concentrated in tropical regions. South America has the highest diversity of Asteraceae in the world, yet taxonomic and distributional knowledge gaps remain. This study compiles an updated catalog of Asteraceae native to South America, based on national and regional checklists and ongoing large-scale flora projects. The resulting checklist includes a total of 6,940 species and 564 genera native to South America to date, which represent about a quarter of the family’s global diversity. Countries already considered to be megadiverse show the greatest diversity, such as Brazil with 2,095 species, followed by Peru (1,588), Argentina (1,377), and Colombia (1,244), with this diversity mainly focused on the Brazilian Highlands and the Andes. Species endemism also peaks in Brazil, but Sørensen distances reveal the Chilean flora to be eminently different from the rest of the continent. Tribes better represented in the continent are Eupatorieae, Senecioneae and Astereae, also with a remarkably presence of entirely South American subfamilies representing earliest diverging lineages of the Asteraceae, such as Barnadesioideae, Wunderlichioideae, Famatinanthoideae, and Stifftioideae. It is estimated that the discovery and description curves have not yet stabilized, and the number of species is likely to increase by 5 to 10% in the coming years, posing major challenges to continental-scale conservation.

## Introduction

Cataloguing the estimated 20% of the undescribed plant life before it is lost has never been more urgent (Joppa et al., 2011; Grace et al., 2021). The United Nations Convention on Biological Diversity’s Global Strategy for Plant Conservation proposed five main objectives: understanding, documentation and recognition of plant diversity, its effective conservation, and its sustainable use and public awareness (United Nation’s Convention on Biological Diversity, 2011).

These objectives are difficult to address due to what is known as “plant awareness disparity”, associated with urban societies’ disconnection from plants (Parsley, 2020). The disconnection is difficult to cope with, given persistent and significant knowledge gaps and shortcomings in the sense of taxonomic, distributional and phylogenetic information (Hortal et al., 2015). These gaps become even more evident when trying to assess conservation efforts on a continental scale (Habel et al., 2013), as is the case of South America. The human footprint is rapidly expanding in the continent (Zalles et al., 2021), leading many ecosystems and species to be classified as threatened.

Efforts to scientifically document the floristic diversity of South America have been continuous since the 17th Century, including renowned expeditions by naturalists such as Alexander von Humboldt, Aimé Bonpland, Georg Markgraaf, Alexandre Ferreira, Louis Feuillée, Carl Friedrich Philipp von Martius and Charles Darwin, to name a few. Also, European naturalists and botanists that settled in the Americas, contributed significantly to botanical knowledge, as in the case of José Celestino Mutis, Rodulfo Amando Philippi and José Cuatrecasas. Several botanical communities developed strongly with the advent of modern times, mainly in Argentina, Brazil, Colombia, and Peru.

The late 20th century set the stage for important regional checklists like Tropicos (https://www.tropicos.org/) led by the Missouri Botanical Garden, the BDG program to document the flora of the Guianas or Mesoamerican Flora. These initiatives have promoted accuracy and authoritative knowledge in taxonomy and nomenclature in the Neotropics, reflected in multiple species lists, herbarium specimens confirming locations, and regional checklists and floras with reliable information.

Tropicos initiative revealed the presence of almost 125,000 vascular plant species in the Americas, from which 82,000 are from South America (Ulloa Ulloa et al., 2017). According to this evaluation, Brazil holds the most diverse vascular plant flora, with more than 33,000 species, followed by Colombia with around 24,000 species, and Peru with around 19,000 species. As reference in North America: Mexico, another megadiverse country, harbors almost 23,000 species. These numbers are not yet stabilizing, with a description rate of 744 species per year over the last 25 years (Ulloa Ulloa et al., 2017).

The daisy family (Asteraceae) is considered as the world richest plant family and has a global diversity of between 25,000 and 30,000 species ranging from the polar circles to the Equator (Funk et al., 2009; Palazzesi et al., 2022). According to the last global account (Panero and Crozier, 2016), at the global scale diversity peaks in South America (6,316 species), followed by Asia (6,016 spp.), North America (5,404 spp.), Africa (4,631 spp.), Europe (2,288 spp.) and Australasia (1,444 spp.)

The family has been recognized as the second most diverse vascular plant family in the Americas with ca. 12,000 species, after Orchidaceae (ca. 13,000) and ahead Fabaceae (7,000), with Brazil, Colombia and Peru being the richest countries in Asteraceae species (Ulloa Ulloa et al., 2017).

**Figure 1:**
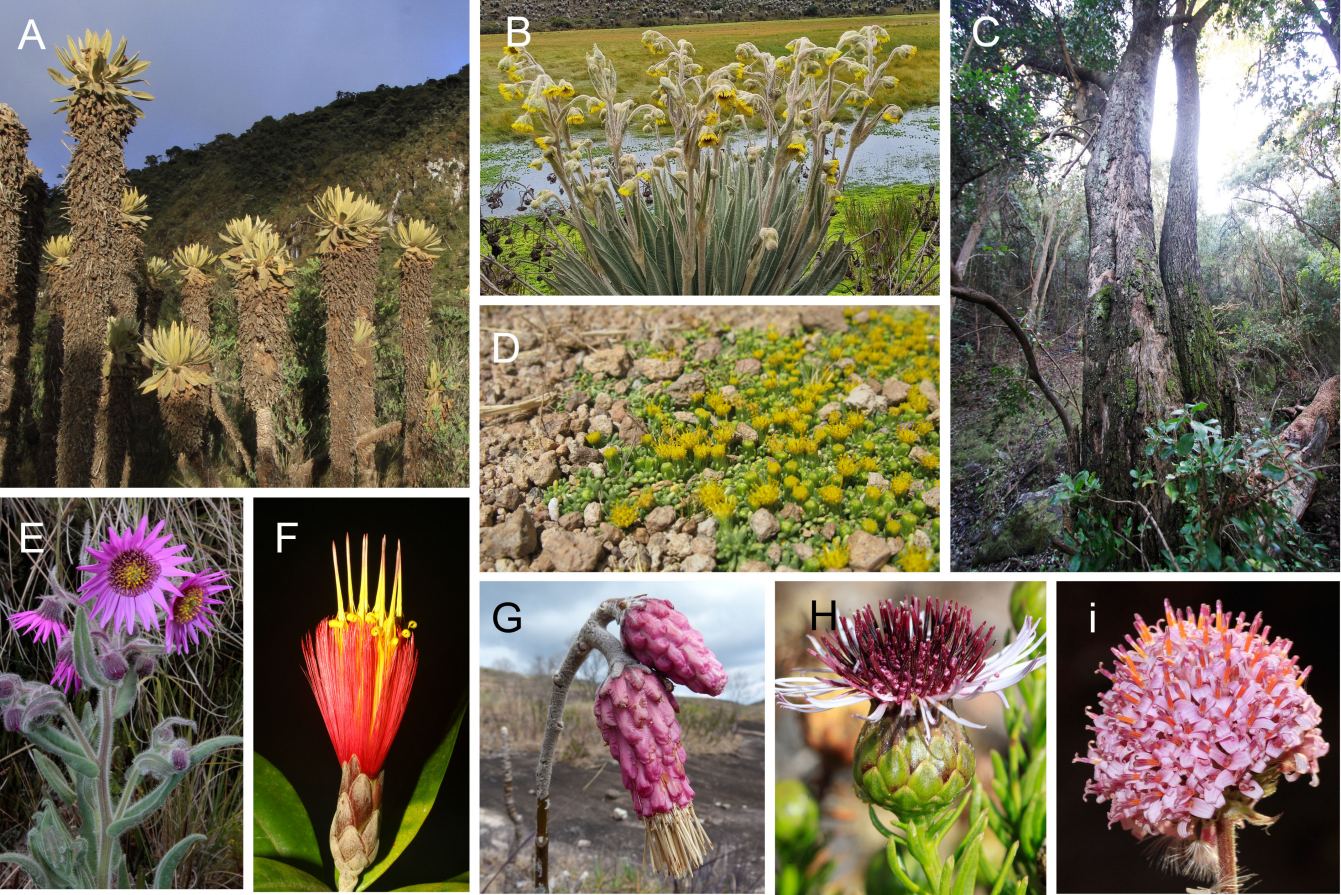
Diversity in growth form and floral morphology in representatives of the Asteraceae across South America: **(A)** *Espeletia hartwegiana* Sch. Bip. ex Cuatrec., from the Andean Páramos; **(B)** *Espeletia summapacis* Cuatrec., from the Colombian Páramos; **(C)** tree species *Archidasyphyllum excelsum* (D. Don) Cabrera, from coastal central Chile; **(D)** *Senecio humillimus* Sch. Bip. ex Wedd., creeping species from the Central Andes; **(E)** *Senecio formosoides* Cuatrec., representative of the most species-rich genus at the continental scale; **(F)** *Stifftia fruticosa* (Vell.) D.J.N. Hind & Semir, Brazilian representative of the subfamily Stifftioideae; **(G)** *Wunderlichia insignis* Baillon, an endemic species from Brazilian inselbergs and representative of the subfamily Wunderlichioideae; **(H)** *Gypothamnium pinifolium* Phil., representative of a monospecific genus from Atacama; **(I)** Inflorescence in Central Andean *Polyachyrus sphaerocephalus* D. Don. [photo credits: F.A. Avila, M. Monge, A. Moreira-Muñoz].

Asteraceae have undergone a series of dispersals and explosive radiation events since they appeared in the late Cretaceous (Viviana et al., 2015; Mandel et al., 2019), and are currently major components of subtropical biomes worldwide (Funk et al., 2009), showing all possible growth forms from trees to herbs, including shrubs, vines, succulents and specific forms such as the rosette trees from Juan Fernández Islands. A key morphological component in the global dispersal of Asteraceae is the pappus, a main part of the floral function of a typical Asteraceae (**Figure 1**). Capitula-like inflorescences are present in related families like Calyceraceae and Goodeniaceae but are not as diverse as in Asteraceae (Zhang and Elomaa, 2021). As phylogenetic knowledge of the family grows and improves, the systematic knowledge at the tribe and subfamily levels increases. Currently 50 tribes are recognized within the family (Susanna et al., 2020) and phylogenetic and biogeographic knowledge is continuously being updated (Heiden et al., 2019; Siniscalchi et al., 2019; Moreira-Muñoz et al., 2020).

As noted, naturalists like José Celestino Mutis (1732–1808) or botanists like José Cuatrecasas (1903–1996) settled in the Americas and contributed significantly to knowledge of the family. Botanists such as Angel L. Cabrera (1908–1999) in Argentina, Graziela Barroso (1912–2003) in Brazil, Leandro Aristeguieta (1923–2012) in Venezuela, or Santiago Díaz-Piedrahita (1944–2014) in Colombia, produced printed checklists and revisions at the country scale and over extensive regions (e.g (Cabrera, 1978)), so that several countries attained adequate knowledge of the family (Katinas et al., 2007).

Through the last decade, several countries assumed the challenge of documenting their floras as a national priority, as was the case with Brazil (BFG (The Brazilian Flora Group), 2018; BFG (The Brazilian Flora Group), 2022), incorporating hundreds of botanists from the region to achieve this goal. Nevertheless, several countries still lack a national catalogue and sampling efforts are still scarce even in most populated areas (Oliveira et al., 2021).

Nevertheless, the continuously growing knowledge has been used as a main source for evaluating numbers of threatened species (e.g (Nakajima et al., 2012)), but the efforts have not been equally performed from one country to another. Additionally, the conservation and sustainable use of native Asteraceae is not of merely botanical interest, but it is in support of ancestral livelihoods and ethnobotanical relations across the continent (e.g (Gutiérrez et al., 2020). They also appear as a target in regional climate change studies (Sklenář et al., 2021).

As a commitment made in several regional botanical meetings, we undertook the challenge of compiling an up-to-date catalog of the Asteraceae at the continental scale. Taking account of species’ description, revision rates, and the specific need for disaggregated information by country to promote continental-level conservation actions, we compiled regional checklists and country level revisions together with the most recent monographs and taxonomic updates (Luebert et al., 2017; Roque et al., 2017; Dillon et al., 2021).

In such a dynamic scientific field, following the assessment by (Ulloa Ulloa et al., 2017), we expect the number of species at the national level to increase by around 50 to 100 species between years 2017 and 2022.

## Methods

### Geographic area

The study area is South America, a continent covering around 17,800,000 km^2^. South America includes 12 countries and one dependent territory: Argentina, Bolivia, Brazil, Chile, Colombia, Ecuador, Guyana, Paraguay, Peru, Suriname, Uruguay, and Venezuela. French Guiana, as an overseas department of France, was included in the analysis, as well as the dependent territories of Malvinas (Falkland) Islands (administered by the United Kingdom and disputed by Argentina). It also included the Galápagos Islands of Ecuador and Chile’s Juan Fernández archipelago in the analysis. Other island territories were not included in the study (see **Supplementary Materials**).

### Catalogue of South American Asteraceae

We compiled the most updated national checklists, floras and taxonomic revisions (See **Supplementary Materials**) to analyze the richness and diversity of Asteraceae in South America. Main reference works are dated from 1990 to 2020. Latest particular distributional o taxonomical works on Asteraceae from South America were included.

We searched for synonyms and taxonomic inconsistencies to integrate this basic national data compilation. We manually checked for all taxa, as well as adding recently described species since the publication of the consulted checklists in IPNI (we closed the search by July 2022).

Taxa were then compiled into one spreadsheet and reviewed for inconsistencies in names, authors, and description dates. We included information about synonyms and the condition of native/endemic. We met regularly online to resolve taxonomic differences and equivocal names. Potential questionable remaining taxa were examined on a case-by-case basis using POWO (Plants of the World Online; https://powo.science.kew.org/), Tropicos (https://www.tropicos.org/home), and the Global Compositae Database (https://www.compositae.org/gcd.php)

We compared our list with Global Compositae Database. 2022. Available at: https://www.compositae.org/gcd.php. (consulted on July 30, 2022) and BFG (2018 onwards).

With a first compilation in hand, we checked with the most authoritative list available so far at the continental scale, which is the “integrated assessment of the vascular plant species of the Americas”, published by (Ulloa Ulloa et al., 2017). The catalogue is available as **Supplementary Dataset 1**.

### Country-level distribution, endemism, and threatened species

We extracted the distribution at the country level from the compiled checklist, including diversity per subfamily and tribe and level of endemism in each country.

Floristic similarity among the 13 countries can be obtained by means of nonmetric multidimensional scaling (NMDS) based on Sørensen distance. For the NMDS analysis, an absence/presence matrix was built with rows (countries) and columns (species). The analysis and graphics were performed using the vegan package in R (see **Supplementary Materials**, **Supplementary Figure 5**).

The graphics depicting tribes by country were generated by enumerating the number of species present in each tribe across South American countries. Using the recorded tribe data for each country, a bar graph was constructed. The resulting images were processed in Illustrator 2020 (ver. v24.3.0, Adobe) (**Supplementary Figures 6A–M**). We added a taxonomic key to the subfamilies occurring in South America.

We searched for “Asteraceae” and “South America” in The IUCN Red List (https://www.iucnredlist.org). The global list is based on the information available for each species at a time. Species that are assessed can be categorized as “threatened” (Critically Endangered, Endangered, or Vulnerable = CR, EN, VU) or “of conservation concern” (including CR, EN, VU, in addition to Near Threatened or Extinct in the Wild (NT, EW) (**Supplementary Tables 5, 6**). For this instance, progresses at national level were not considered, which will be reviewed in future works (Lopez-Gallego et al., 2024).

## Results

**Figure 2:**
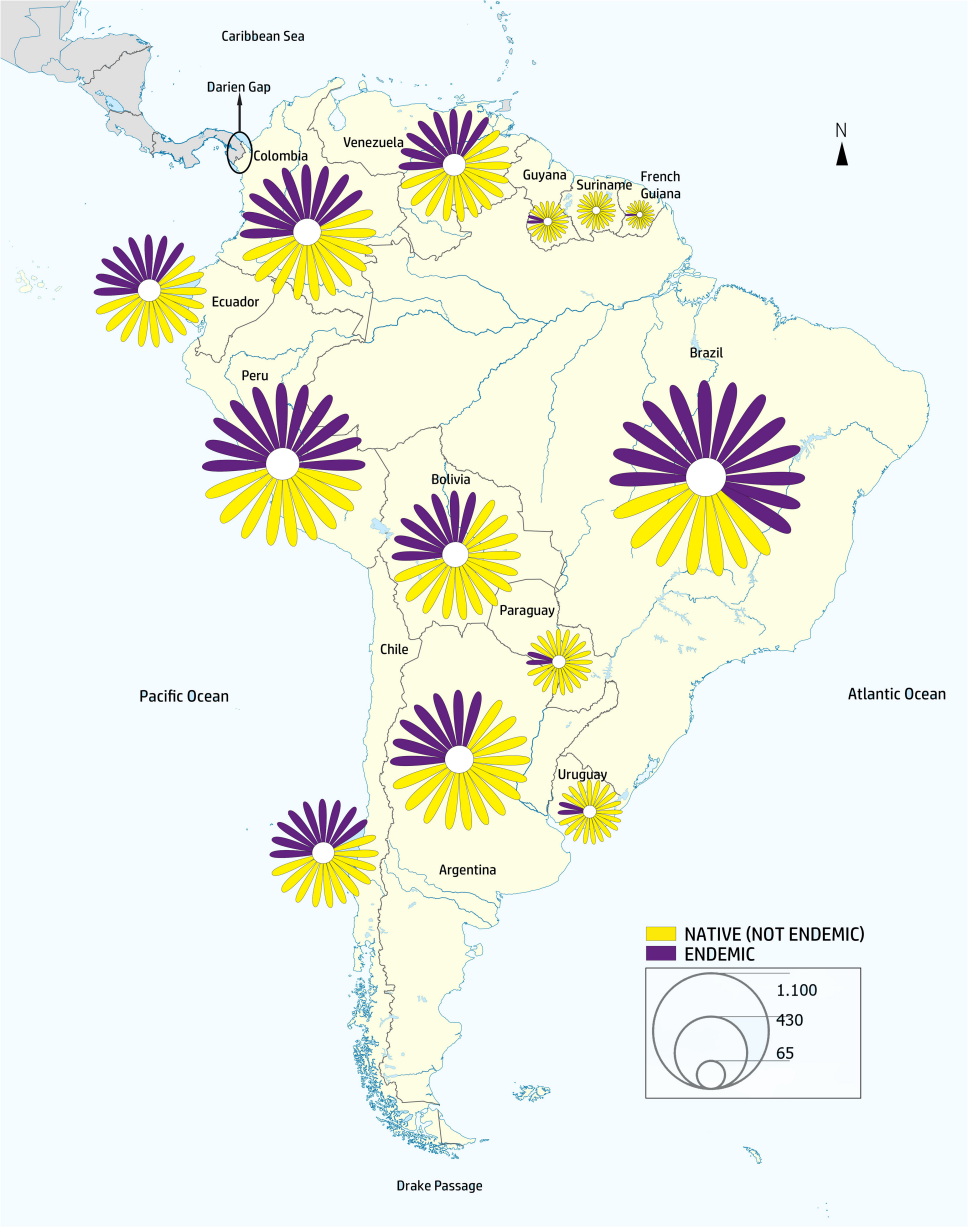
Map of South America representing the diversity of the family across the continent. The map was elaborated with the native/endemic species numbers per country from **Table 1**, by means of ArcGIS Online (www.arcgis.com). The resulting image was processed in Illustrator 2020 (ver. v24.3.0, Adobe). A daisy symbol was used to depict the proportion of each category and its size was adjusted to reflect species proportions present in each country. The size of the symbols represents the number of species per country. Purple represents the proportion of endemic species in each country.

Our verified taxonomic dataset records 6,940 native species and 564 genera of Asteraceae in South America. Brazil has the richest native Asteraceae flora, with 2,095 species, followed by Peru (1,588), Argentina (1,377), and Colombia (1,244) (**Table 1**; **Figure 2**; **Supplementary Dataset 1**).

**Table 1:** Synthesis of the numbers of subfamilies, tribes, genera, species and endemic species in each South American country.

Country	Subfamilies	Tribes	Genera	Native Species	Endemic Species
Argentina	10	27	239	1,377	372
Bolivia	8	24	225	1,192	362
Brazil	9	26	289	2,095	1,354
Chile	6	22	128	826	373
Colombia	7	22	226	1,244	627
Ecuador	7	22	205	935	396
French Guiana	3	10	44	67	4
Guyana	5	14	69	138	1
Paraguay	8	21	138	498	51
Peru	7	23	235	1,588	913
Suriname	3	11	41	65	0
Uruguay	8	21	102	329	16
Venezuela	8	23	200	793	323

Native South American species are grouped into 564 genera represented in the flora. The most species-rich genera are the widely distributed *Senecio* (652 spp, Senecioneae), *Baccharis* (378 spp, Astereae), *Mikania* (377 spp, Eupatorieae), *Pentacalia* (189 spp, Senecioneae), and *Stevia* (148 spp, Eupatorieae) (**Supplementary Dataset 1**). Most genera are represented by fewer than 20 species and are endemic to one or two countries (**Supplementary Figure 2**).

Of the 6,940 native Asteraceae species in South America, a large proportion (69%; or 4,794 species) is endemic to a single country, while only eight species occur in 12 countries. None of the species is found in all countries and territories. In turn, Brazil is the country with the largest number of endemic species, 1,353, followed by Peru (913) and Colombia (627) (**Table 1**; **Supplementary Table 2**; **Supplementary Dataset 1**).

Considering only endemic species by the respective country’s area, the most endemic-rich country is Ecuador (14.3 spp/10,000 km^2^), followed by Peru (7.1 spp/10,000 km^2^) (**Supplementary Figures 3, 4**). The genus *Mikania* (Eupatorieae) has the largest number of endemic species by country, with 137 endemic species in Brazil.

Floristic similarity among the 13 countries analyzed has been approached by means of nonmetric multidimensional scaling (NMDS) (**Supplementary Figure 5**). Brazil shares most of its flora with Paraguay and Uruguay, while the Guyanas appear closer together. Ecuador, Colombia and Peru show commonality among their florae, while Chile appears as isolated.

The family is represented by 30 of 50 currently recognized tribes (APG IV, 2016; Susanna et al., 2020). The richest tribe is Eupatorieae, with 1,448 species, followed by Senecioneae with 1,270 and Astereae with 875 (**Supplementary Table 1**). Each country has different proportions of representatives of tribes within the family (**Supplementary Figure 6**).

The most species-rich subfamily is Asteroideae including 16 tribes and 5,235 species (**Supplementary Tables 2–4**). Other subfamilies are represented by one to three tribes, with a remarkably presence of entirely South American subfamilies representing earliest diverging lineages of the Asteraceae, such as Barnadesioideae, Wunderlichioideae, Famatinanthoideae, and Stifftioideae. The country with the highest presence of subfamilies and tribes is Argentina, with 10 subfamilies and 27 tribes represented in its flora (**Table 1**; **Supplementary Tables 2, 3**).

### Key to South American subfamilies of Asteraceae

Key based on (Anderberg et al., 2007; Katinas and Funk, 2020) and own observations.

1. Florets with simple, uniseriate, 3-celled hairs (barnadesioid trichomes) … Barnadesioideae
1’. Florets without such hair, usually with many hair types or glabrous … 2
2. Florets with corolla bilabiate, subbilabiate, ligulate, tubular deeply lobed; style branches short or long, with rounded papillae or glabrous … 3
3. Style with papillae wider than tall or glabrous … 4
4. Style with papillae wider than tall, composed of cobblestone-like units comprising several epidermal cells … Famatinanthoideae
4’. Style usually glabrous, when papillose composed of single, slightly protruding epidermal cells … 5
5. Capitula homogamous, florets with corolla tubular; apical anther appendiges apiculate … Gochnatioidea
5’. Capitula homogamous or heterogamous, florets with corolla bilabiate, tubular or marginal bilabiate and central tubular; apical anther appendiges acute … Stifftioideae
3’. Style with papillae longer than width … 6
6. Florets with corolla bilabiate, or bilabiate or subbilabiate and tubular; style with papillae only on branches, sometimes below the bifurcation, or with papillae at the apices; papillae simple with 1 epidermal cell … Mutisioideae
6’. Florets with corolla tubular; style with papillae on the branches and shaft; papillae multi-seriate with 2 or 3 epidermal cells … Wunderlichioideae
2’. Florets with corolla tubular, deeply or shortly lobed, ligulate, or marginal ligulate and central tubular, rarely reduced or absent; style branches short or long, with acute papillae or hairs, covering the branches, sometimes below the bifurcation, at the apices or with a ring of papillae below the bifurcation, rarely glabrous, sometimes with long papillose sterile appendages … 7
7. Florets with corolla tubular deeply lobed, ligulate, or marginal ligulate and central tubular; all florets or central florets with style branches long and slender, free or fused leaving the apices free, with a ring of acute papillae below the bifurcation or dorsally covering the branches to below the bifurcation … 8
8. All florets with style branches long and slender, fused leaving the apices free, with a ring of acute papillae below bifurcation (carduoid style) … Carduoideae
8’. All florets or central florets with style branches long and slender, free or fused leaving the apices free, with acute papillae dorsally covering the branches to below the bifurcation (vernonioid style) … 9
9. Abundant milky latex; capitula homogamous, florets with corolla ligulate … Cichorioideae
9’. With or without milky latex; capitula homogamous or heterogamous, florets with corolla tubular deeply lobed, or marginal ligulate and central tubular … Vernonioideae
7´. Florets with corolla tubular shortly lobed, or marginal ligulate and central tubular, rarely reduced or absent; all florets, central florets, staminal florets or pistilate florets with style branches long or short, with acute papillae or hairs at the apices, rarely with papillae dorsally covering the branches and shaft, rarely glabrous, sometimes with long papillose sterile appendages … Asteroideae

### Threatened status assessment

Specific conservation assessments are very diverse across the continent. To obtain a global vision, we checked species that have been assessed according to IUCN criteria (www.iucnredlist.org). In this sense, 777 taxa of Asteraceae (only 11.2% of the total) have been assessed across the continent: 436 can be considered “threatened” (i.e. “Critically Endangered”, “Endangered” or “Vulnerable”) (**Supplementary Table 5**). Other 339 species are categorized as “Near Threatened”, “Least Concern”, or “Data Deficient” (**Supplementary Table 5**). The country with the highest number of threatened species (238) is Ecuador, followed by Colombia (119 species) (**Supplementary Table 6**). For Colombia, other 390 species are under assessment (Lopez-Gallego et al., 2024). On the one hand, this reflects an increase in land use changes and a retreat in native forests, and on the other the phenomenon that more species have been assessed in Ecuador and Colombia, which is not the case in other countries.

Our analysis also found 177 non-native or introduced species. The countries with the largest numbers of documented introduced species are Argentina (109), Chile (104) and Brazil (87), mainly in subtropical latitudes with the highest demographic concentration and ecosystem alterations. Most introduced species pertain to the Asteroideae (92 species) and to the tribes Cichorieae, Anthemideae, and Cardueae (**Supplementary Tables 7, 8**). This issue reflects the long history of exchange through maritime trade, mainly with European countries, in addition to cultural links from colonial times to the present.

### Description rates

Our compilation shows that the species description and recircumscription rate has not shown a stabilization trend. The description rate differs from one country to another. Over the past 30 years, between six and 100 species have been described (or resurrected) in different countries. The most prolific taxonomists describing South American Asteraceae are J. Cuatrecasas, with 812 names (basionyms or recombinations); while R. M. King and H. Robinson published 708 names (**Supplementary Table 9**; **Supplementary Dataset S1**). In total, names are assignable to 488 taxonomists that described species along the continent.

Countries like Brazil and Colombia are considered megadiverse and are among the world’s leading countries for the continued discovery of new species. 

## Discussion

This is the first attempt to catalog the diversity of Asteraceae in South America by means of a comprehensive checklist. We were able to check inconsistencies between national checklists in the last 30 years, while adding the new taxa described and combined in the last five years, since the report by (Ulloa Ulloa et al., 2017).

South America holds the world greatest diversity of Asteraceae species, corroborating the findings of (Panero and Crozier, 2016). Since the last integrated assessment (Ulloa Ulloa et al., 2017), the number of species increased by 9.5% in Venezuela, 5% in Peru, 4.8% in Brazil and 3.6% in Colombia. Countries like Brazil, Colombia, and Peru are among the world’s leading countries in terms of the numbers of new species discovered per year (Cheek et al., 2020). This is also the case with Ecuador and Bolivia, which show less improvements due to shortcomings in floristic update efforts. All these differences could be explained by difficulties reported by (Panero and Crozier, 2016) to find taxonomic treatments for some genera in large tribes for a worldwide compilation, so our task was facilitated due the more limited scope of our searches. There may be some differences regarding the concept of different species, which is inherent to the taxonomic task (ter Steege et al., 2019).

The four most speciose countries -Brazil, Peru, Argentina, and Colombia- account for 47% of the total species richness of the continent, earning them the category of megadiverse countries (Mittermeier et al., 1997). Asteraceae is one of the major components of the flora of important bioregions in these countries, such as the tropical and subtropical Andes, Páramos, Cerrado, Campos Rupestres, Atlantic Forest, Pampas, and Tepuis (BFG (The Brazilian Flora Group), 2018; Colli-Silva et al., 2019). Future integrated checklists should address the biodiversity for each bioregion. Additionally, mainly based on the new “Flora do Brasil” effort, an estimation of the numbers to be added over the next 30 years, and considering megadiverse Peru, Ecuador, Colombia and Bolivia, allows one to estimate an increase of between 5% and 10% in total numbers by 2050 (**Supplementary Figure 7**).

Threatened or near-threatened species include many range-restricted and endemic species whose distribution ranges have been reduced due to the advance of the human footprint along the continent (Zalles et al., 2021). The prevalence of non-native or introduced species in the subtropical latitudes reflects the long history of exchange through maritime trade, mainly with European countries, in addition to cultural links from colonial times to the present (Monge et al., 2016; Gutiérrez et al., 2020).

Helping society to overcome current “plant awareness disparity” includes the need for updated knowledge of diversity in broad territories to inform continental-scale conservation actions. Our analyses of diversity of South American Asteraceae make it clear that this is an exceptionally rich flora of broad interest for conservation attention and investment.

The trend of discoveries in the family reinforces the importance of improving sampling efforts at a continental level to close knowledge gaps and better evaluate threatened status. South America is politically and culturally very complex and diverse and has a rapidly growing human footprint, along with limited botanical literacy. It is still a territory prone to new discoveries by plant taxonomists and parataxonomists to work in priority areas supporting community-based conservation initiatives. By adding taxonomic evidence, we provide a better baseline for people from across the continent to further engage with our biocultural heritage and the livelihoods supporting landscape-scale regenerative actions.

## Conclusion

Our updated catalogue of Asteraceae native to South America, comprises a total of 6,940 species and 564 genera native to South America to date, which represent about a quarter of the family’s global diversity. Countries that show the greatest diversity are Brazil (2,095 species), Peru (1,588), Argentina (1,377), and Colombia (1,244). Asteraceae is one of the major components of the flora of important bioregions in these countries, such as the tropical and subtropical Andes, Páramos, Cerrado, Campos Rupestres, Atlantic Forest, Pampas, and Tepuis. All these bioregions show a rapidly growing human footprint, posing major challenges for continental-scale conservation.

## Author contributions

AM-M: Conceptualization, Formal analysis, Funding acquisition, Investigation, Methodology, Project administration, Resources, Supervision, Validation, Visualization, Writing – original draft, Writing – review & editing. MM: Conceptualization, Data curation, Investigation, Methodology, Resources, Validation, Writing – original draft. MG: Conceptualization, Funding acquisition, Investigation, Methodology, Validation, Writing – original draft, Writing – review & editing. FA: Conceptualization, Data curation, Investigation, Validation, Writing – original draft, Writing – review & editing. VM-F: Data curation, Formal analysis, Methodology, Validation, Visualization, Writing – original draft. GH: Data curation, Investigation, Validation, Writing – original draft. BB: Data curation, Investigation, Validation, Writing – original draft. SB: Data curation, Investigation, Validation, Writing – original draft. JN: Data curation, Investigation, Supervision, Validation, Writing – original draft. VS: Formal analysis, Software, Visualization, Writing – original draft. JR-C: Formal analysis, Software, Visualization, Writing – original draft. DG: Data curation, Formal analysis, Funding acquisition, Investigation, Methodology, Supervision, Validation, Writing – original draft, Writing – review & editing.
