# Supplementary material for: South America holds the greatest diversity of native daisies (Asteraceae) in the world: an updated catalogue supporting continental-scale conservation
Source: Front Plant Sci. 2024 May 30;15:1393241. doi: 10.3389/fpls.2024.1393241 (PMC11169850; doi:10.3389/fpls.2024.1393241)
Supplement: Supplementary file 2 [file DataSheet_2.docx]

Supplementary Materials for

**South America holds the greatest diversity of native daisies (Asteraceae) in the world: an updated catalogue supporting continental-scale conservation**

Andrés Moreira-Muñoz, Marcelo Monge, Mariana A. Grossi, Fabio Ávila, Vanezza Morales-Fierro, Gustavo Heiden, Berni Britto, Stephan Beck, Jimi N. Nakajima, Vanina G. Salgado, Juan Facundo Rodríguez-Cravero, Diego G. Gutiérrez

* Corresponding authors. Email: [andres.moreira@pucv.cl](mailto:andres.moreira@pucv.cl); [digutier@macn.gov.ar](mailto:digutier@macn.gov.ar)

**The PDF file includes:**

Figs. S1 to S7

Tables S1 to S9

Basic Bibliography by country

**Other Supplementary Materials for this manuscript include the following:**

Dataset S1: Species of Asteraceae of South America

**Geographic area of the study**

The study area is South America, a continent covering around 17,800,000 km^2^, bordered by the Pacific Ocean to the west, mainly the Atlantic Ocean (especially the Caribbean Sea to the northwest) and Central America to the north, the Atlantic Ocean to the east, and the Atlantic and Pacific Oceans (Drake Passage) to the south. South America’s only land boundary is the Darién Gap, a geographic region within Central America, between the North and South American continents, shared by Colombia and Panama.

Island territories not included in the study are: the ABC islands (Aruba and Curaçao) as autonomous countries, and Bonaire as a Dutch municipality; the archipelago of San Andrés, Providencia, and Santa Catalina in the Caribbean (Colombia), Easter Island and Sala and Gómez islands in Oceania (Chile), and the subantarctic South Georgia and South Sandwich Islands (administered by the United Kingdom and disputed by Argentina) were not included in our study. Trinidad and Tobago is usually considered as part of the West Indies in the Caribbean and was omitted from our analysis. Despite Ascension Island (dependency of Saint Helena, Ascension and Tristan da Cunha, a British Overseas Territory) and Bouvet Island (dependency of Norway), and sometimes Panama, having also been considered parts of South America on occasion, they were not included in this work.

### Checklist of South American Asteraceae

Asteraceae stands out as one of most diverse families in the Americas, including mega-diverse countries like Colombia and Mexico. A synthesis came with the comprehensive assessment by (Ulloa Ulloa et al. 2017). for the entire Americas. In this assessment, Asteraceae appeared as the most species-rich family in the Southern Cone (Argentina, Chile, Uruguay, Paraguay and southern Brazil) (15%), North America (14%), and Mexico (13%); being the second in species richness in Central America and tropical Andean countries (Ulloa Ulloa et al. 2017).

**Our verified taxonomic dataset** **records 6,940 native species and 564 genera of Asteraceae in South America**. **Brazil has the richest native Asteraceae flora, with 2,095 species, followed by Peru (1,588), Argentina (1,377), and Colombia (1,244)** (Table 1, Fig. 2, **Fig S1, Data S1, Data S2).**


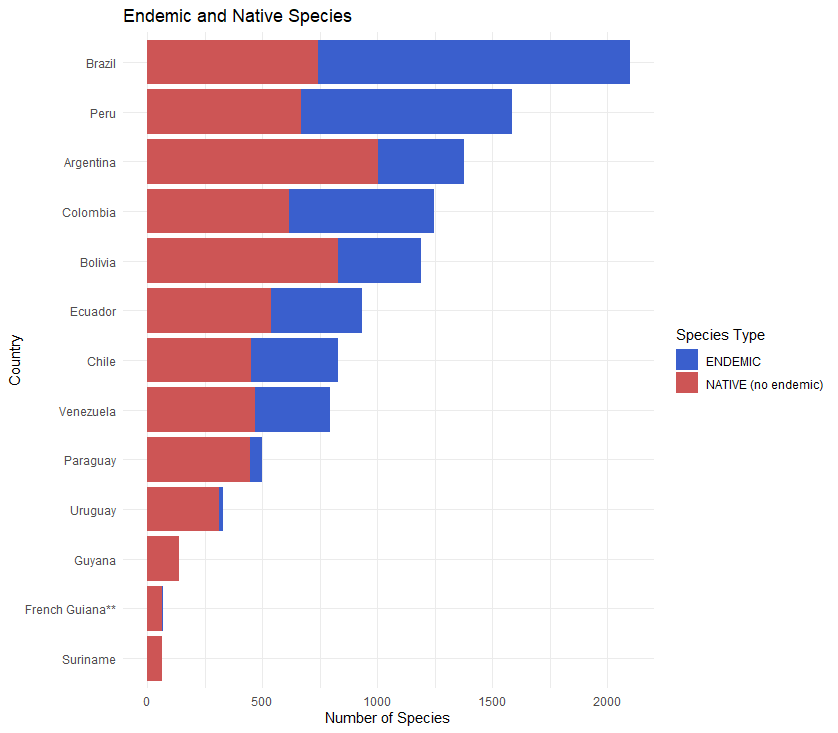


Fig. S1.

Total number of Asteraceae in each South American country. Blue portion of each bar are endemic species for the respective country.

**Fig. S2.**

Number of genera versus number of species. Most genera (192) in South America are represented by only one species (monospecific). Few genera are represented by more than 100 species. Only the genus *Senecio* is represented by 652 species along South America.


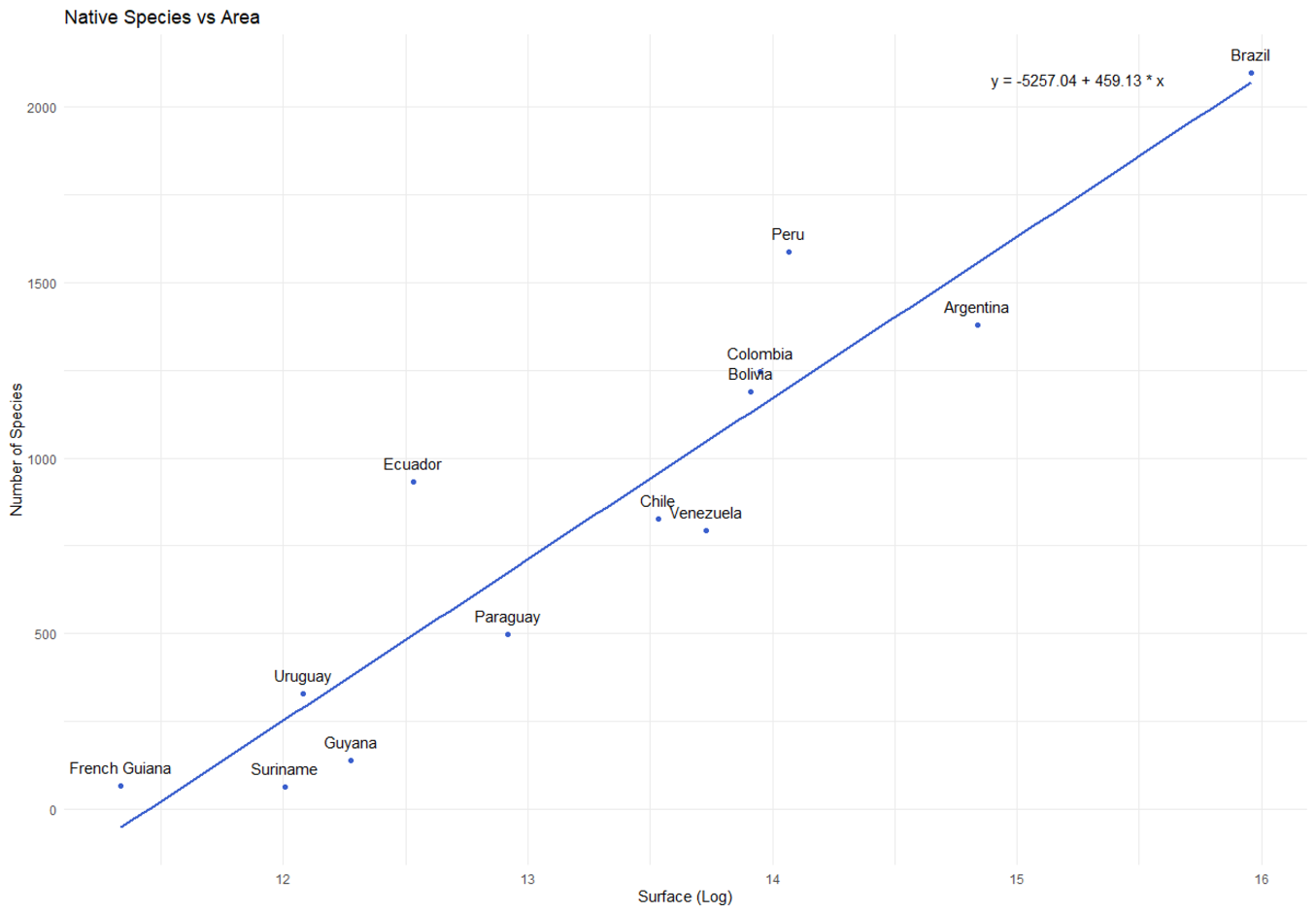


Fig. S3.

Number of species corrected by area (X axis represents x 10,000 square km).


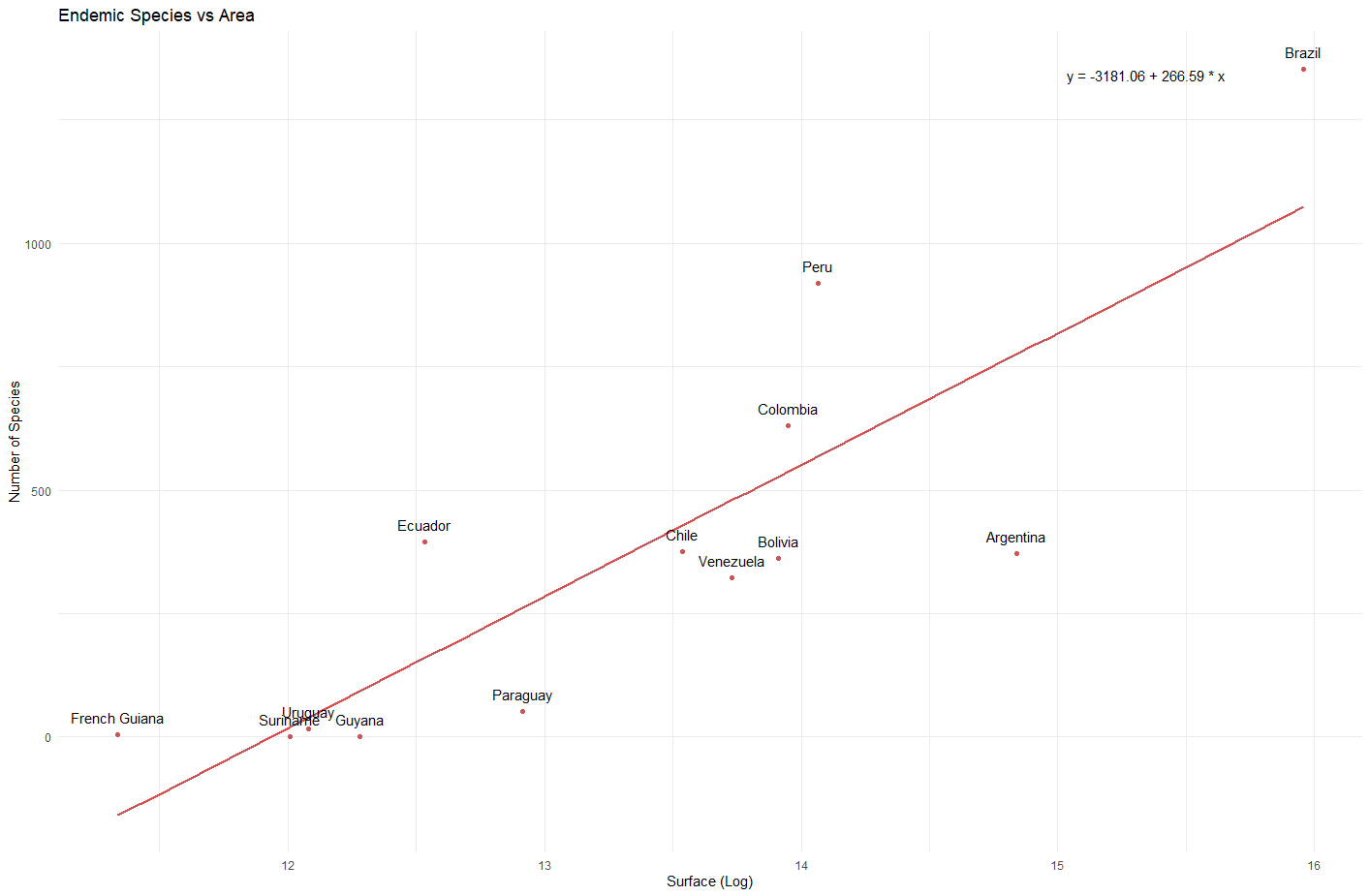


Fig. S4.

Number of endemic species corrected by area (X axis represents x 10,000 square km).

**
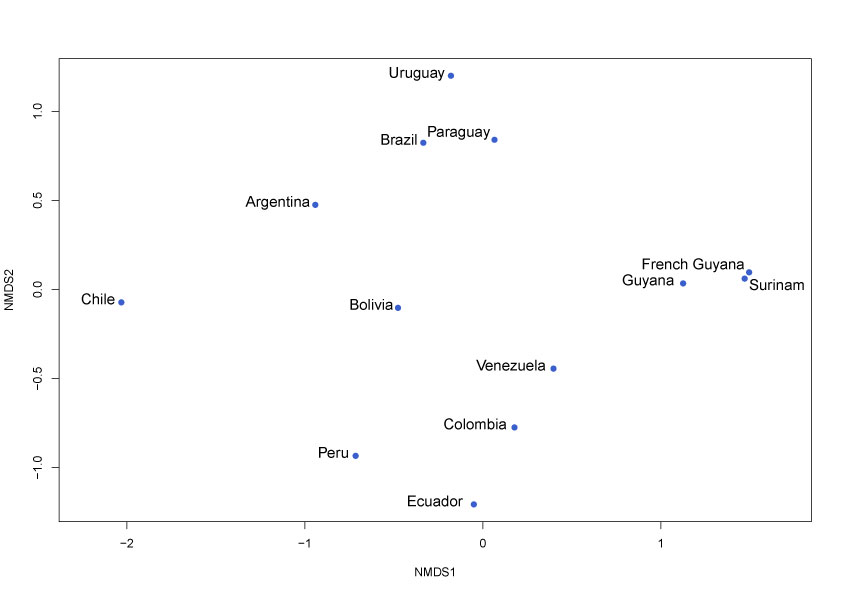
**

**Fig. S5. Floristic similarity among the 13 South American countries**. Similarity of Asteraceae species among countries represented as a nonmetric multidimensional scaling (NMDS). Distance and placement are indicative of similarity among countries. For the NMDS analysis, an absence/presence matrix was built with rows (countries) and columns (species). A distance matrix was built using Sørensen distances. The graphic shows a good fit to the distance matrix, with a stress value of 0.0734 (values below 0.2 are considered a good fit). The non-metric fit R^2 was 0.995, and the linear fit R^2 was 0.968 The analysis and graphics were performed using the vegan package (Oksanen et al. 2022) implemented in R (R Core Team 2021).

Oksanen, J.; Simpson, G.L.; et al. (2022). vegan: Community  Ecology Package. R package version 2.6-2. [https://CRAN.R-project.org/package=vegan](https://cran.r-project.org/package=vegan)

R Core Team (2021). R: A language and environment for statistical  computing. R Foundation for Statistical Computing, Vienna, Austria.  [https://www.R-project.org/](https://www.r-project.org/).

**
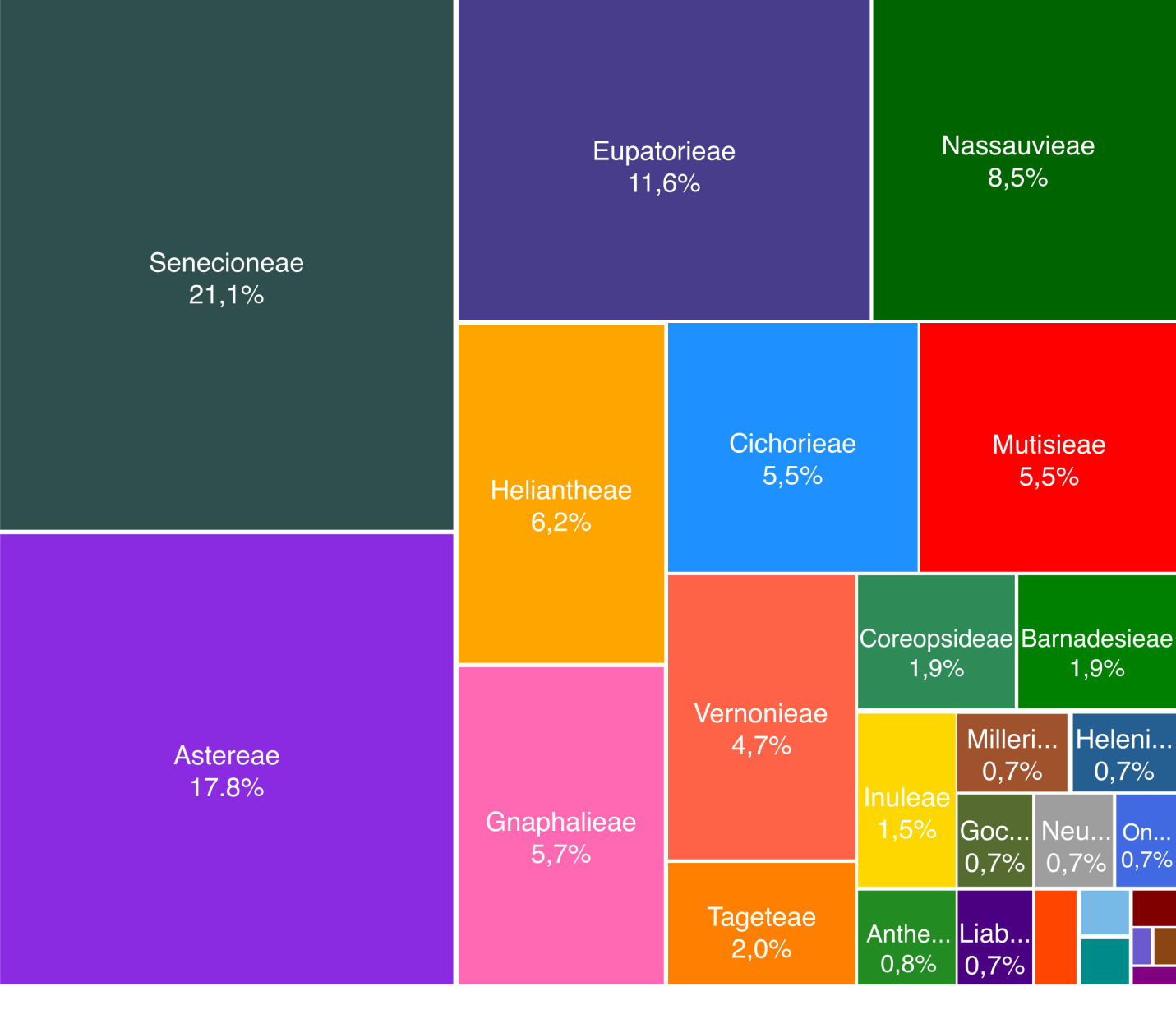
**

**Fig. S6. (A).** Proportions of Tribe representation of Asteraceae in Argentina

**
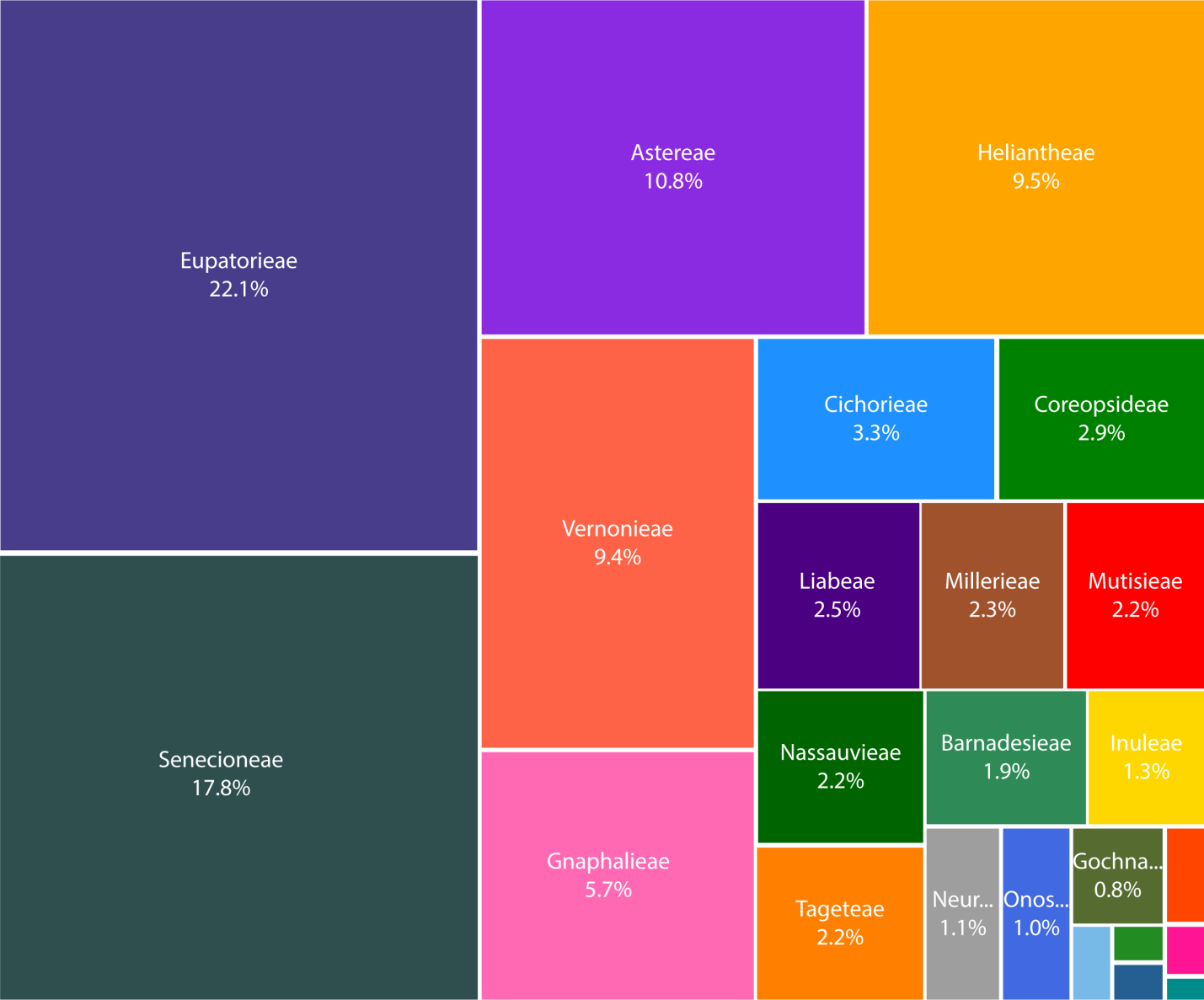
Fig. S6. (B).** Proportions of Tribe representation of Asteraceae in Bolivia

**
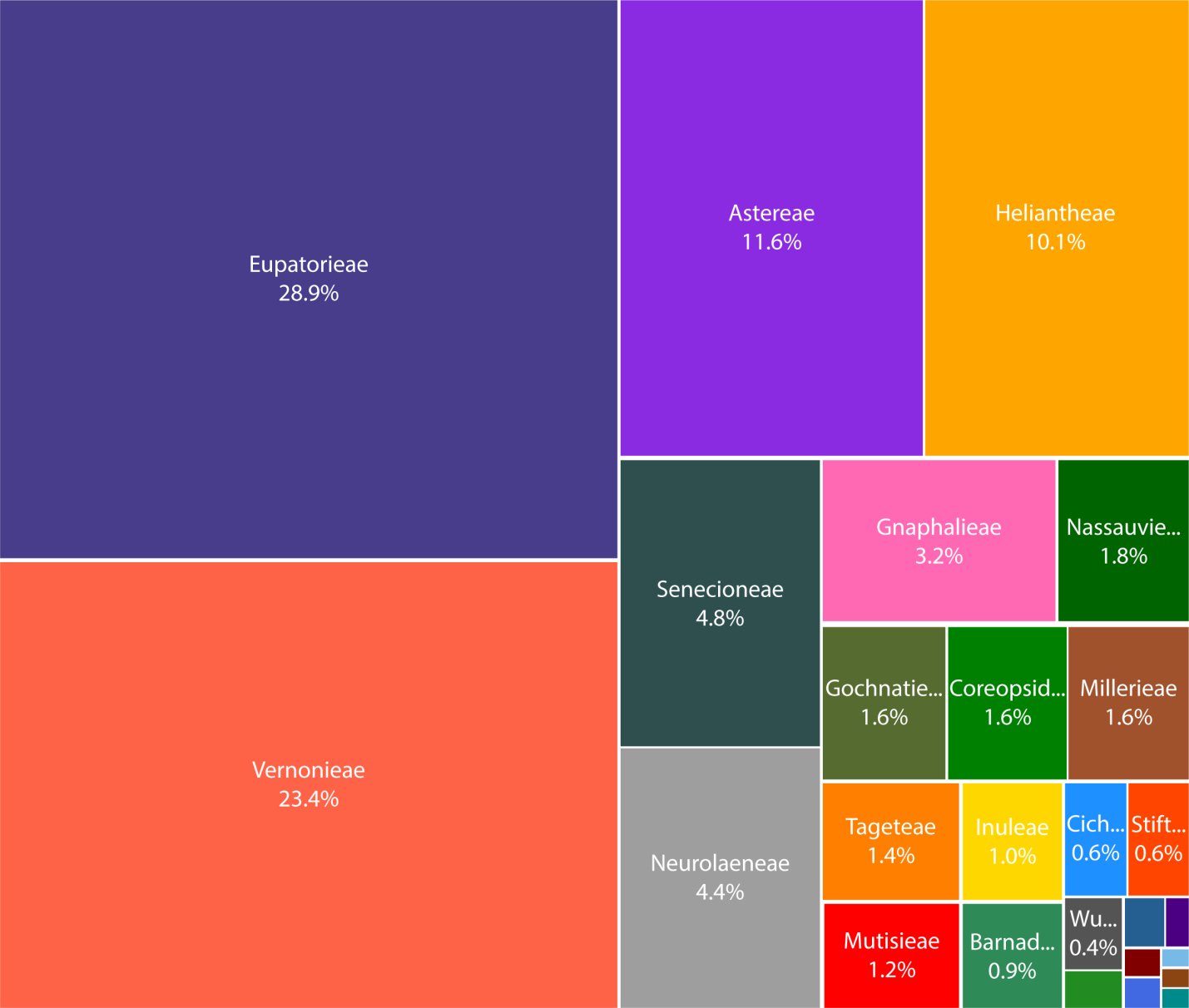
Fig. S6. (C).** Proportions of Tribe representation of Asteraceae in Brazil

**
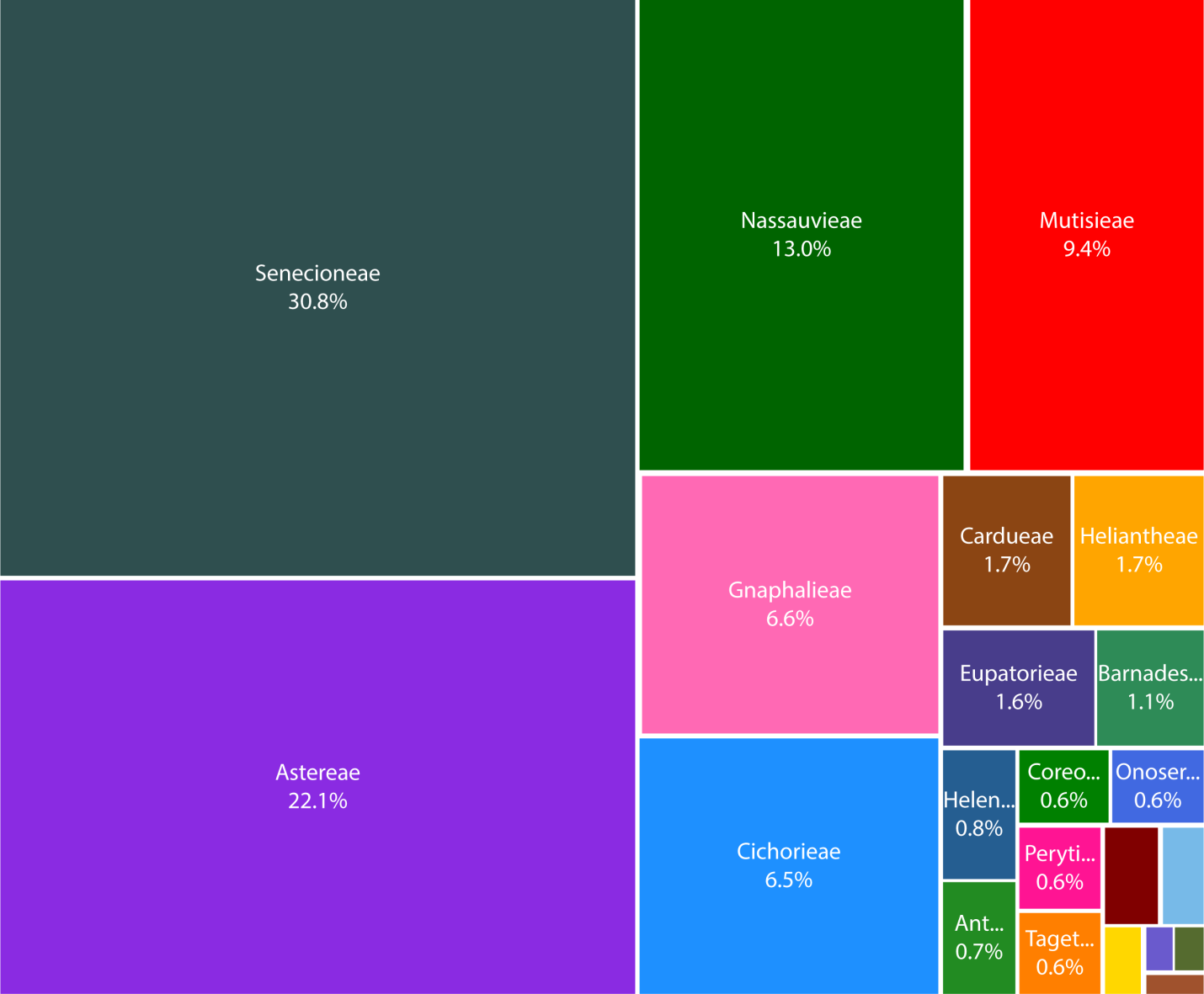
Fig. S6. (D).** Proportions of Tribe representation of Asteraceae in Chile

**
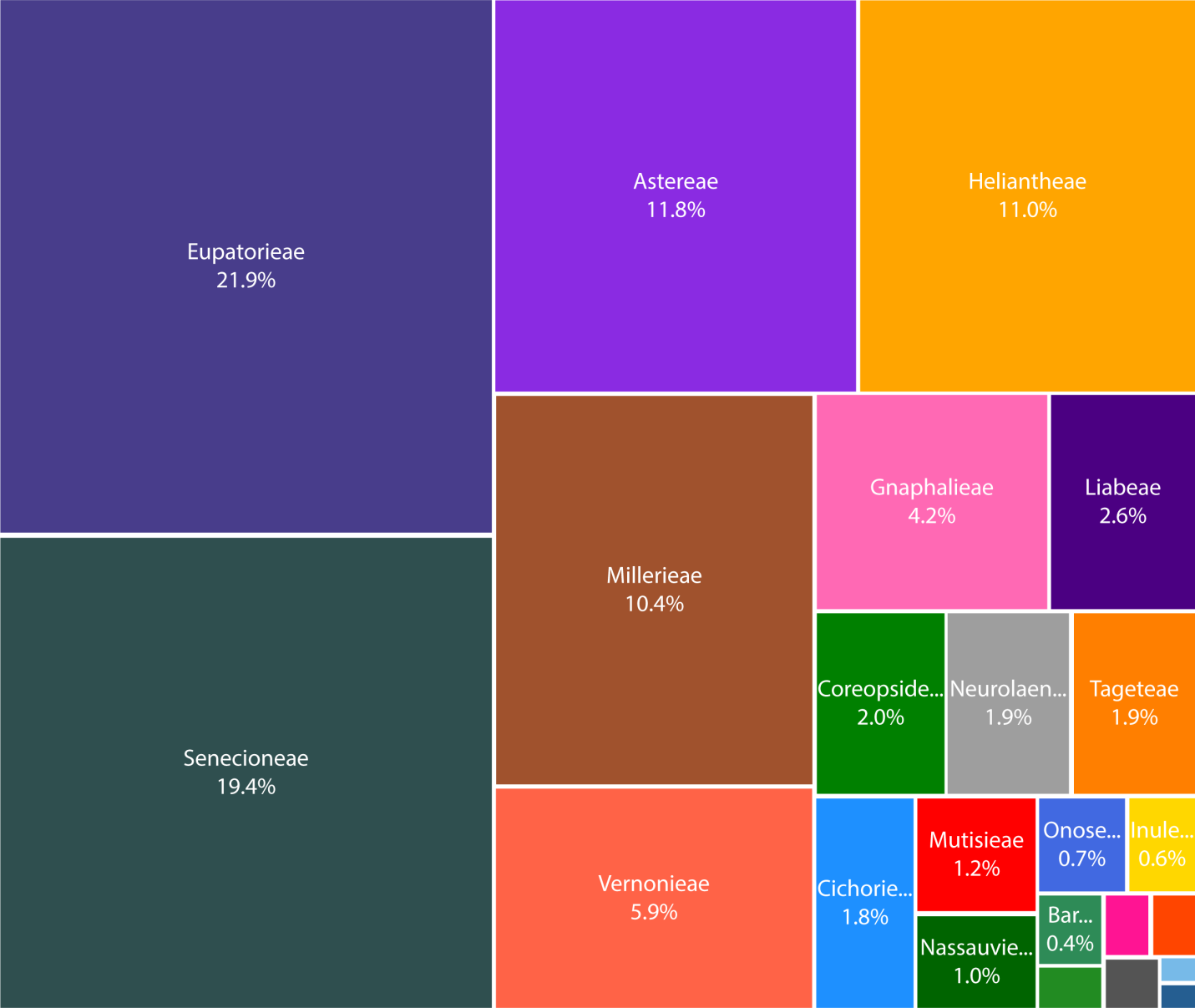
Fig. S6. (E).** Proportions of Tribe representation of Asteraceae in Colombia

**
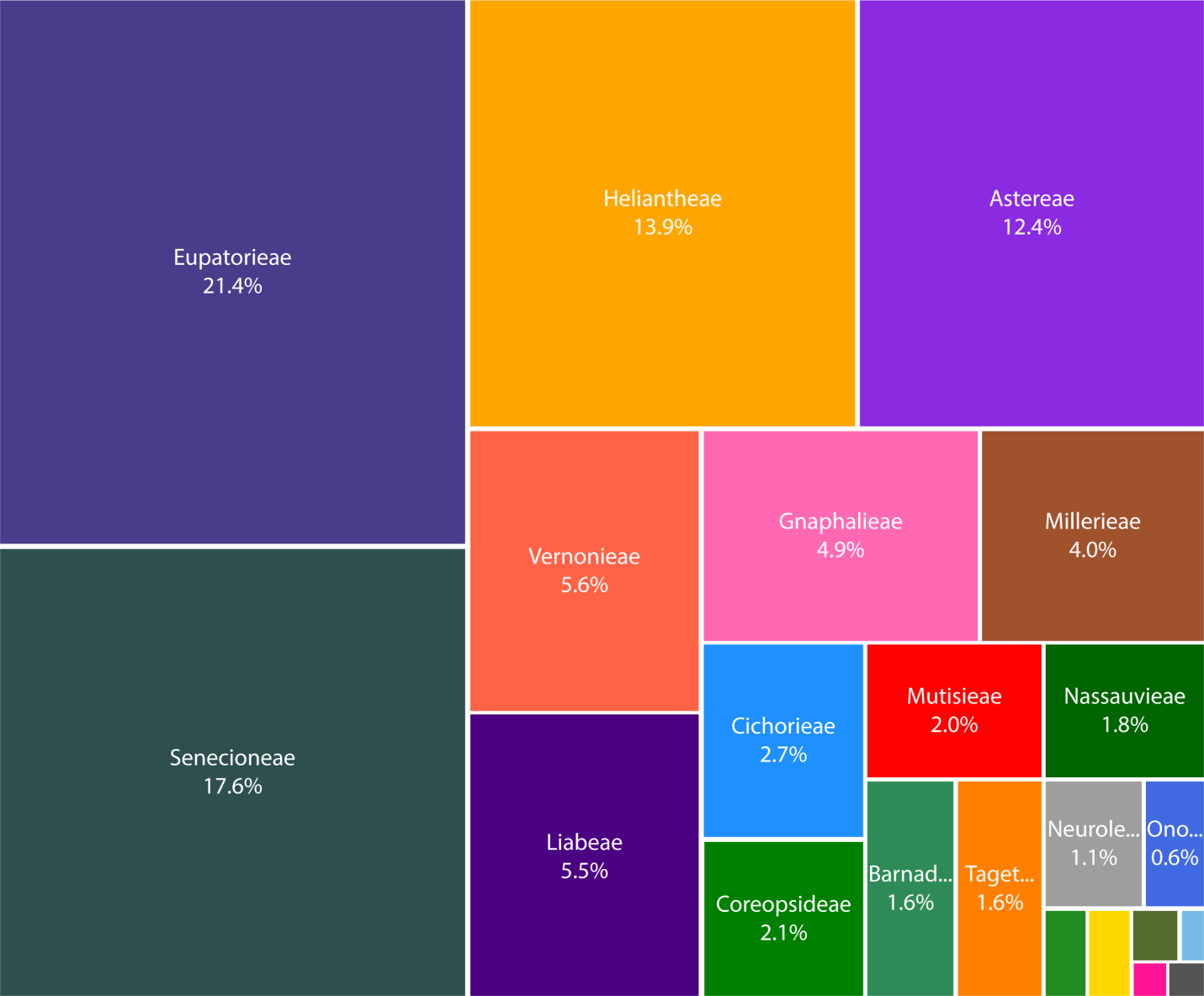
Fig. S6. (F).** Proportions of Tribe representation of Asteraceae in Ecuador

**
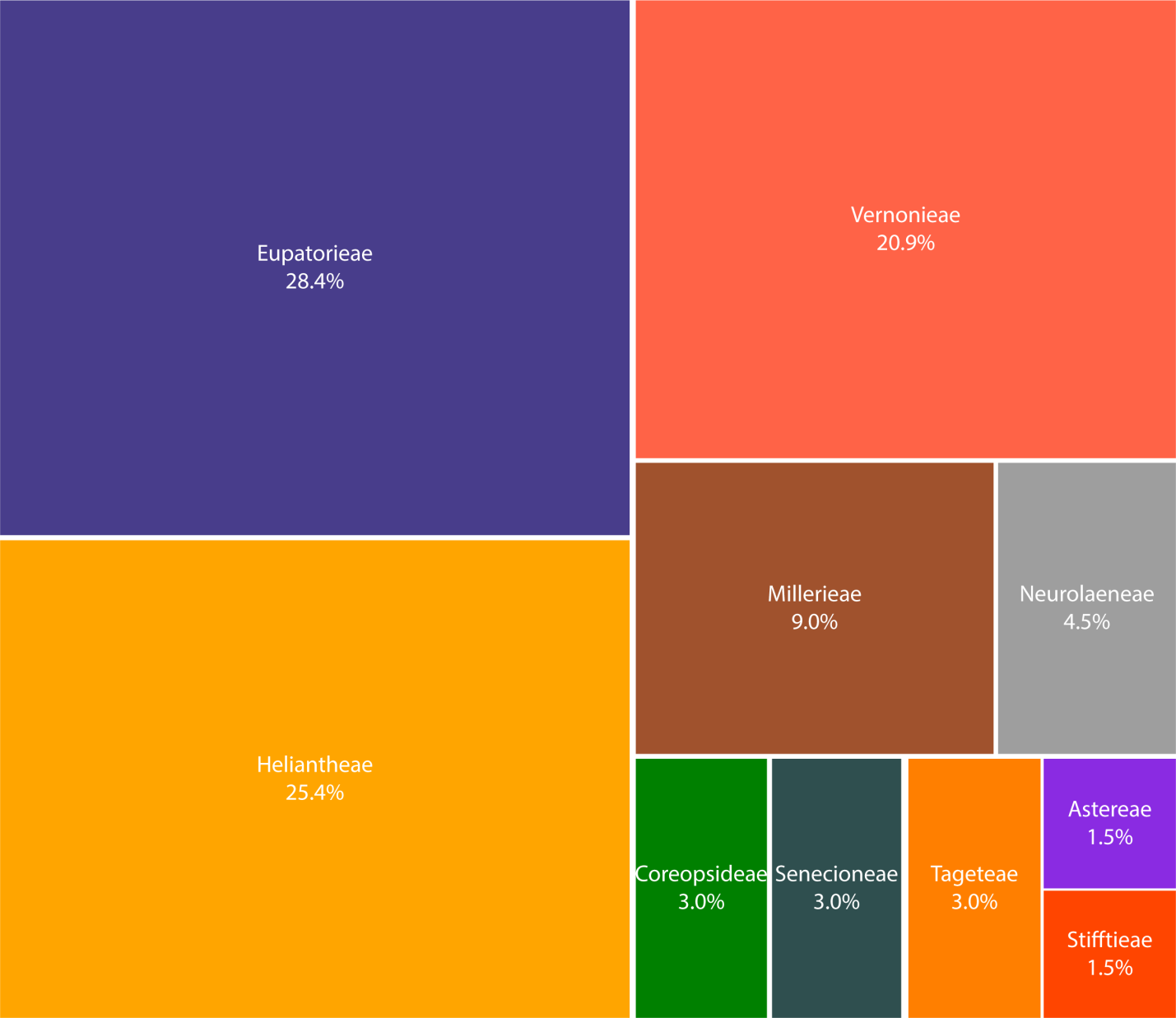
Fig. S6. (G).** Proportions of Tribe representation of Asteraceae in French Guiana

**
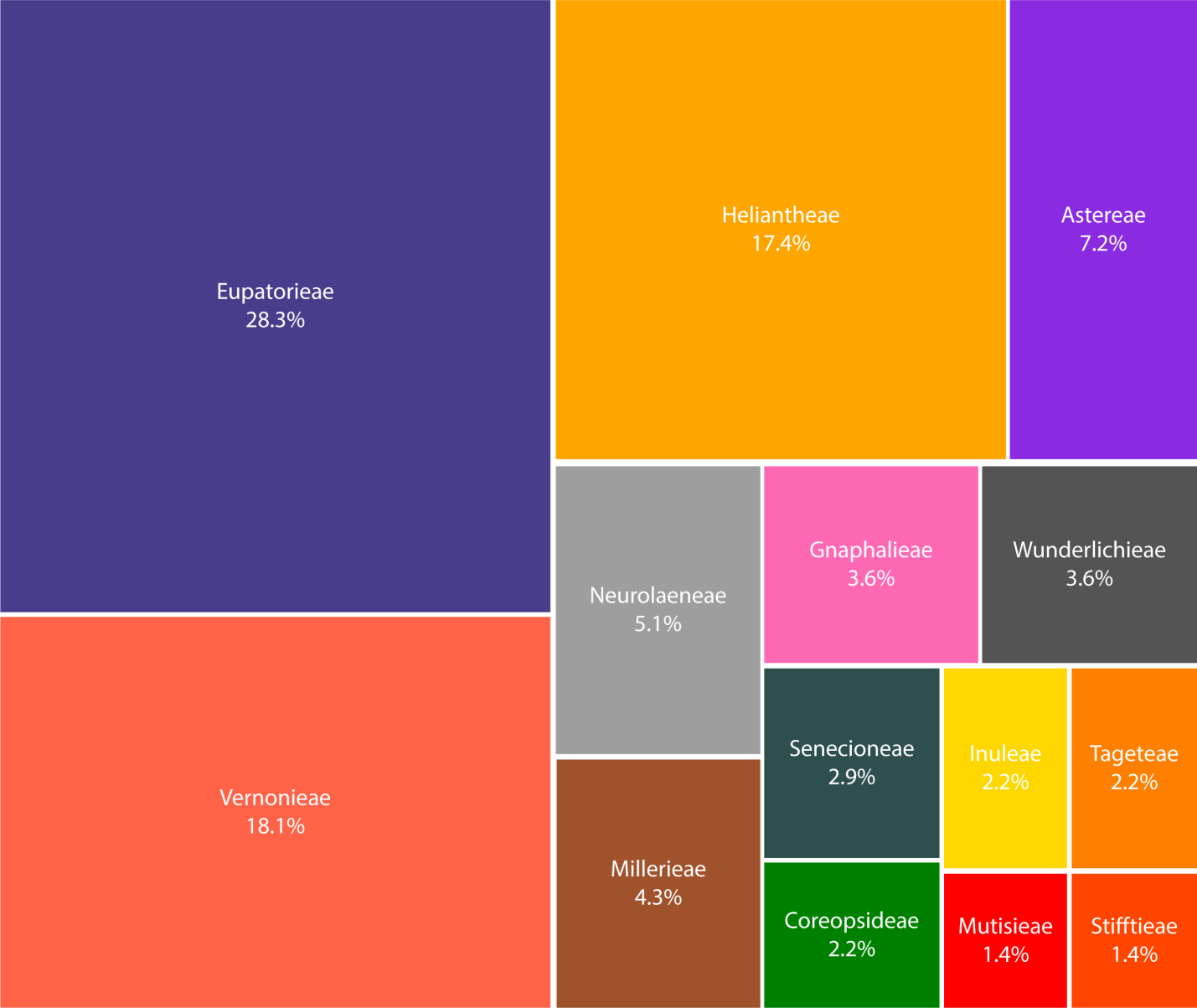
Fig. S6. (H).** Proportions of Tribe representation of Asteraceae in Guyana

**
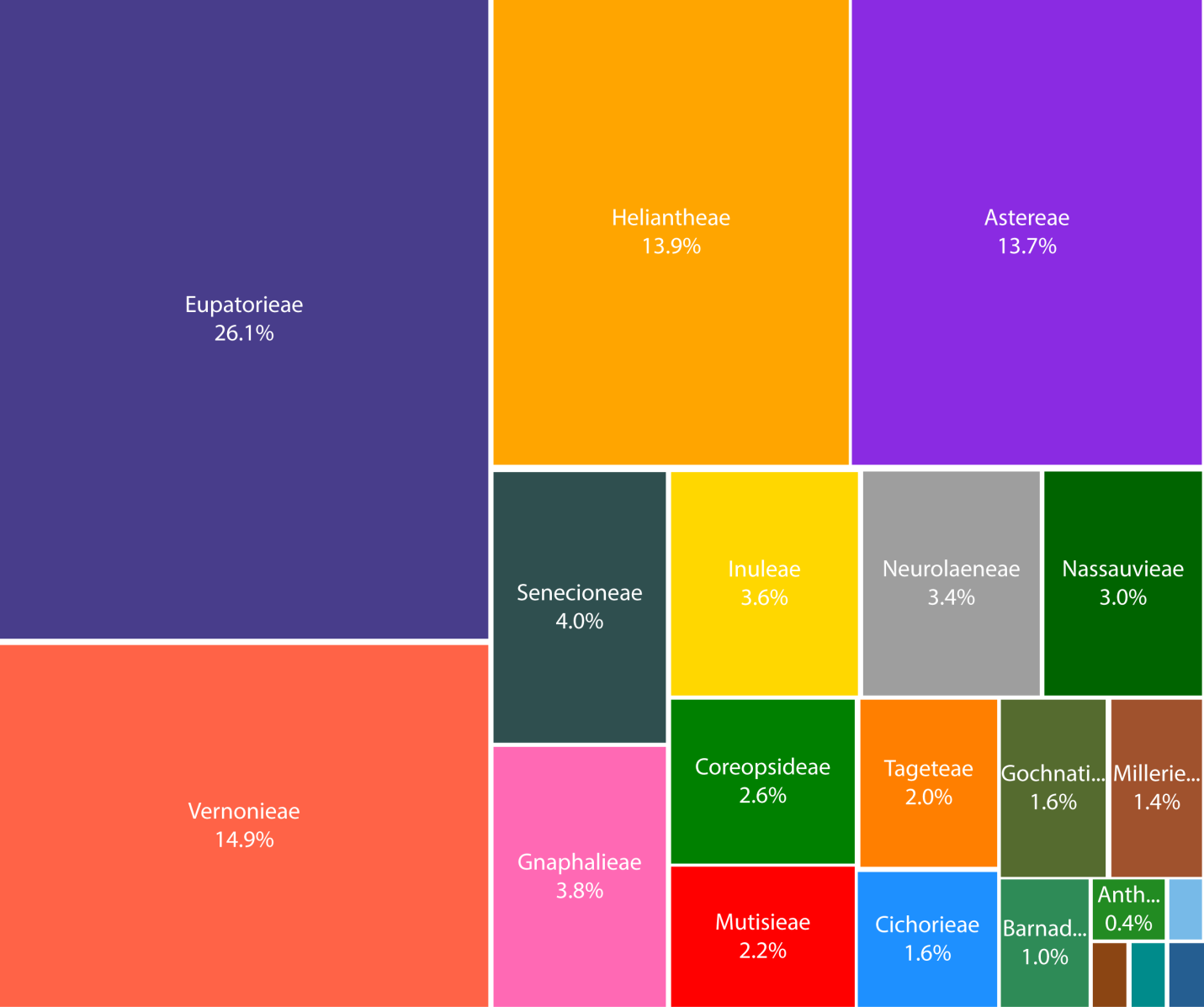
Fig. S6. (I)** Proportions of Tribe representation of Asteraceae in Paraguay

**
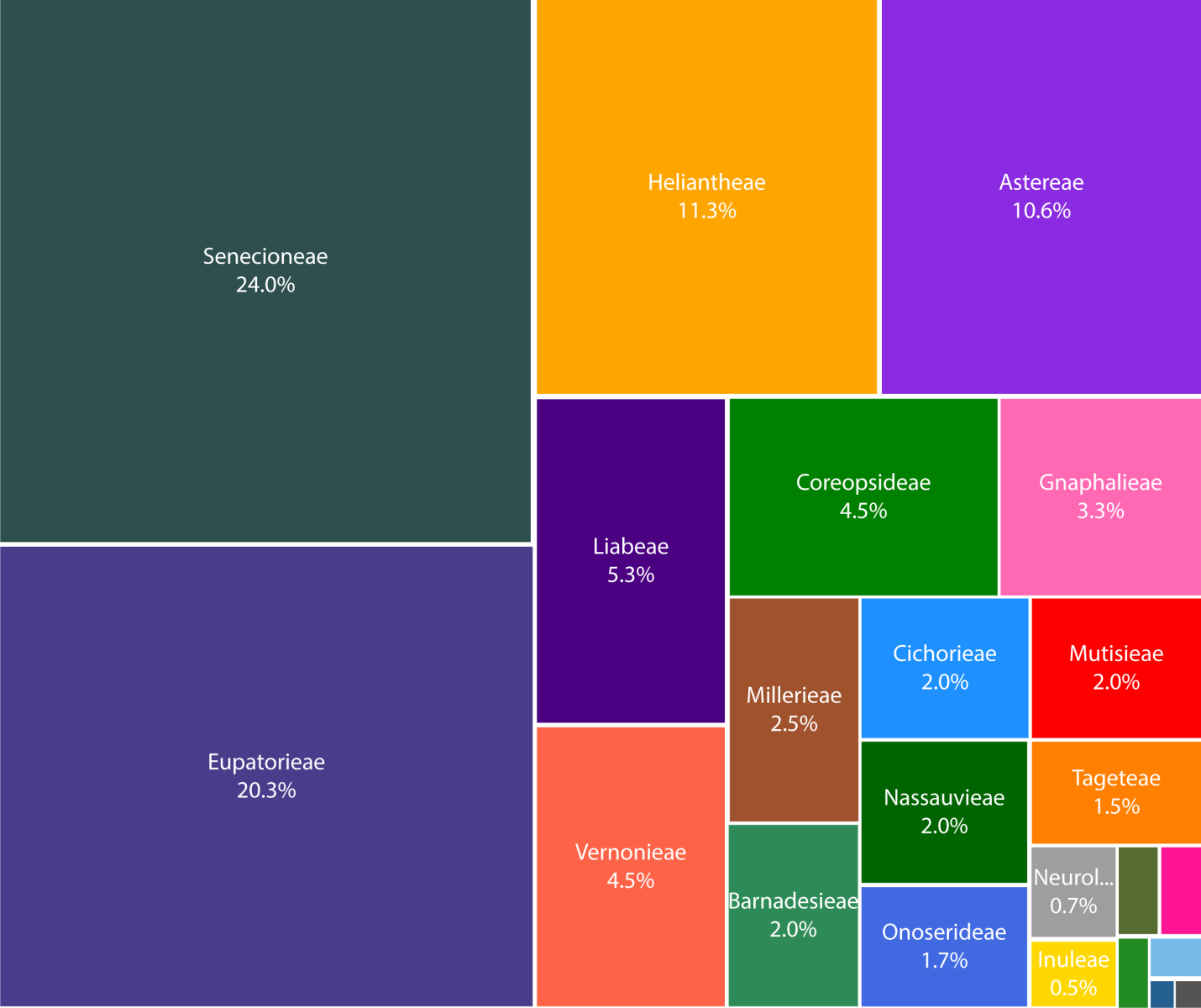
Fig. S6. (J)** Proportions of Tribe representation of Asteraceae in Peru


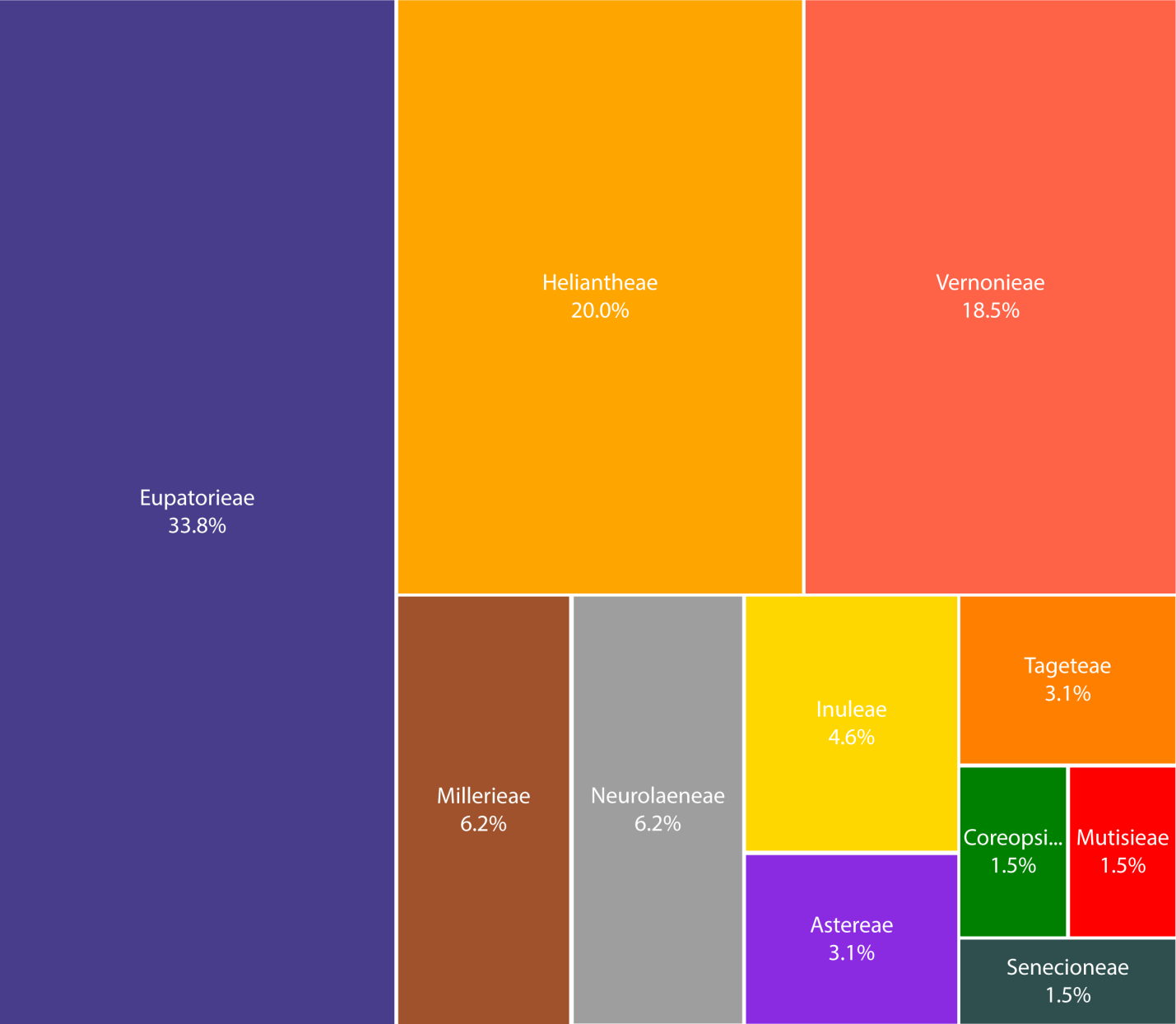


**Fig. S6. (K)** Proportions of Tribe representation of Asteraceae in Suriname


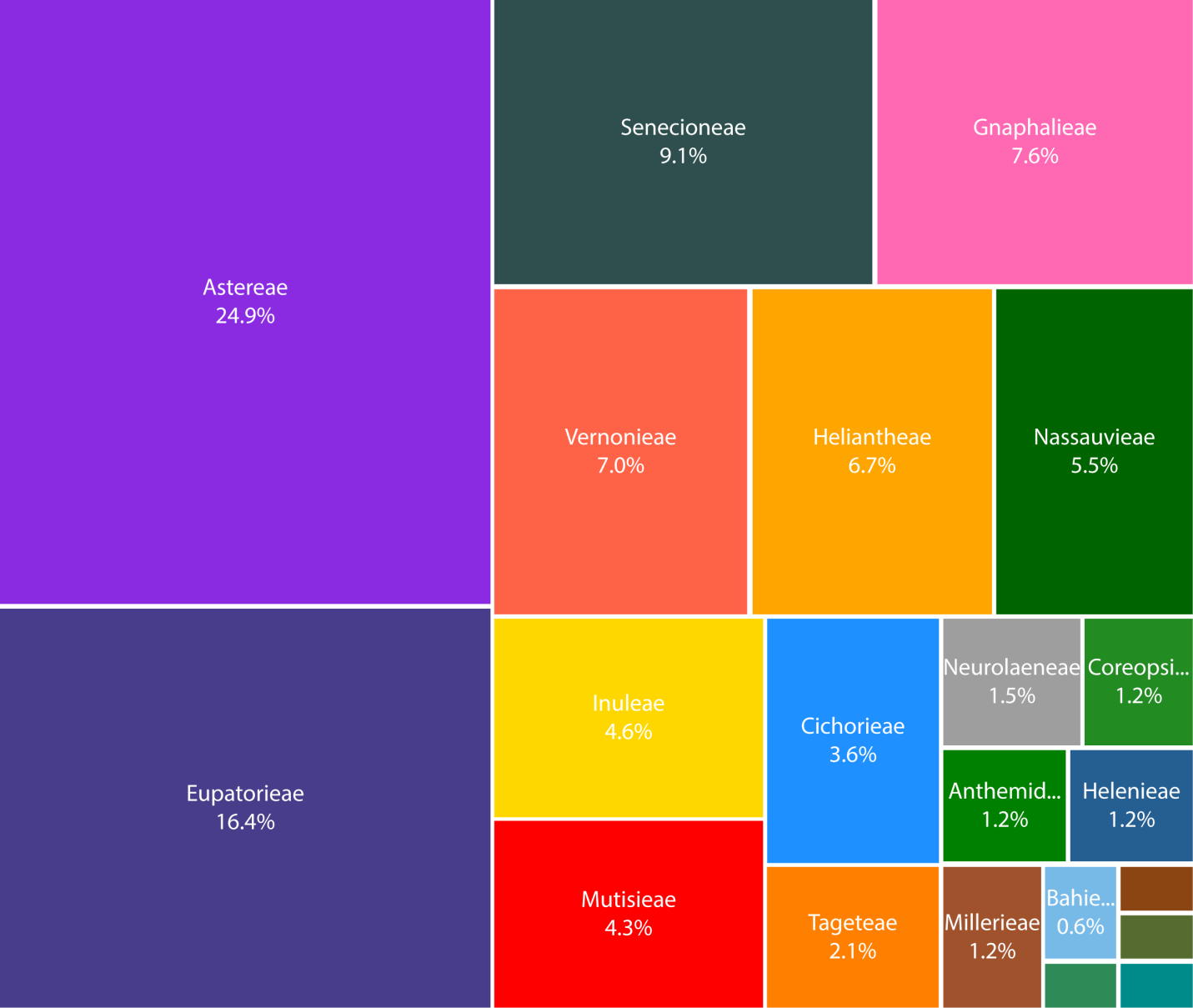


**Fig. S6. (L)** Proportions of Tribe representation of Asteraceae in Uruguay


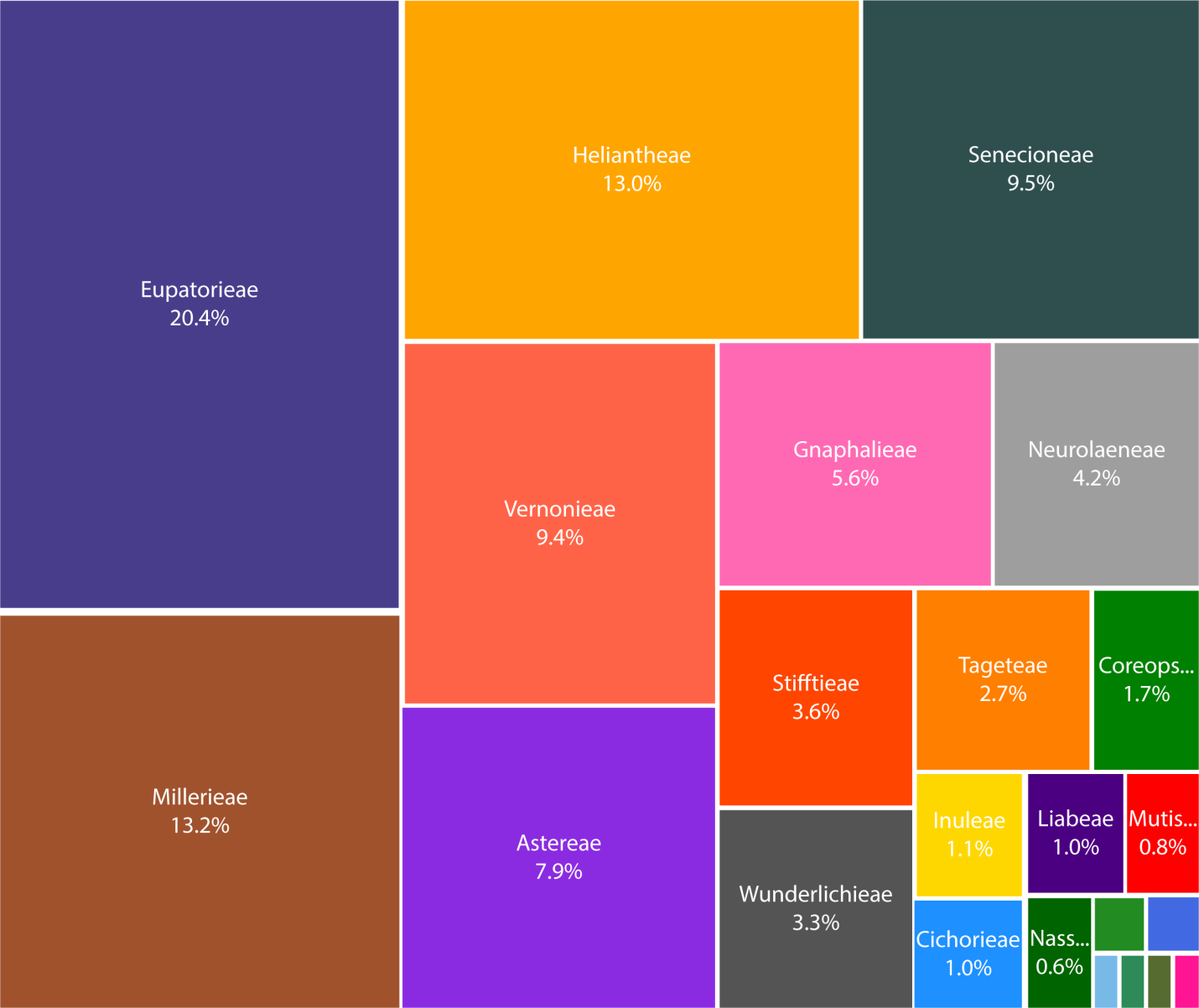


**Fig. S6. (M)** Proportions of Tribe representation of Asteraceae in Venezuela

**
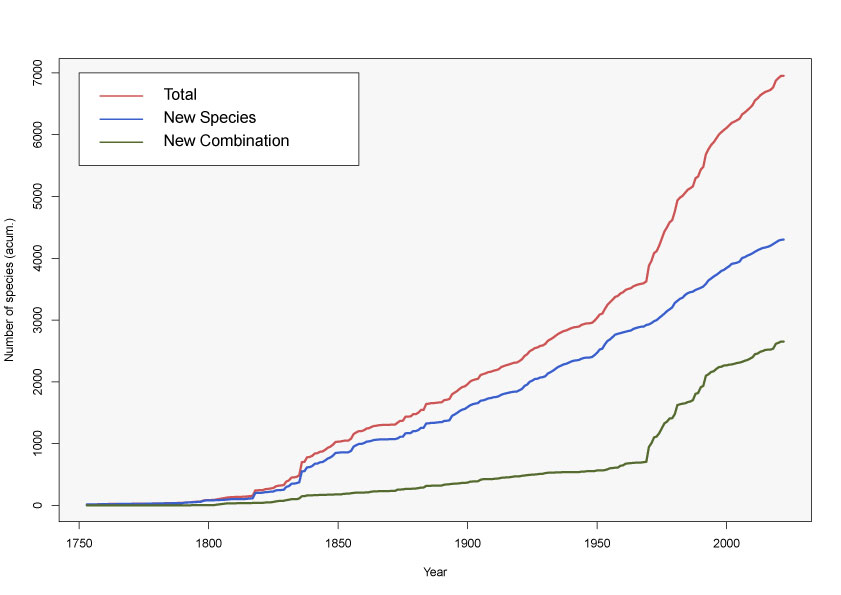
**

**Fig. S7. Accumulation curves of Asteraceae species in South America**. The number of Asteraceae species described per year from 1753 to 2022 for South America. Total numbers in red, new species in blue and new combinations in green line. The graphic was constructed using the cumulative of each category published each year and developed using the ggplot2 package (Wickham et al. 2019) implemented in R (R Core Team 2021).

R Core Team (2021). R: A language and environment for statistical  computing. R Foundation for Statistical Computing, Vienna, Austria.  [https://www.R-project.org/](https://www.r-project.org/).

Wickham, H., Chang, W., & Wickham, M. H. (2016). Package ‘ggplot2’. Create elegant data visualisations using the grammar of graphics. Version, 2(1), 1-189.

Table S1. Species richness by tribe

| No | Tribe | N° species |
| --- | --- | --- |
| 1 | Anthemideae | 12 |
| 2 | Astereae | 875 |
| 3 | Athroismeae | 1 |
| 4 | Bahieae | 5 |
| 5 | Barnadesieae | 95 |
| 6 | Cardueae | 14 |
| 7 | Cichorieae | 174 |
| 8 | Coreopsideae | 130 |
| 9 | Eupatorieae | 1448 |
| 10 | Famatinantheae | 1 |
| 11 | Gnaphalieae | 229 |
| 12 | Gochnatieae | 52 |
| 13 | Helenieae | 19 |
| 14 | Heliantheae | 695 |
| 15 | Hyalideae | 3 |
| 16 | Inuleae | 34 |
| 17 | Liabeae | 131 |
| 18 | Madieae | 4 |
| 19 | Millerieae | 263 |
| 20 | Moquinieae | 2 |
| 21 | Mutisieae | 172 |
| 22 | Nassauvieae | 217 |
| 23 | Neurolaeneae | 146 |
| 24 | Onoserideae | 51 |
| 25 | Perityleae | 10 |
| 26 | Senecioneae | 1270 |
| 27 | Stifftieae | 44 |
| 28 | Tageteae | 92 |
| 29 | Vernonieae | 717 |
| 30 | Wunderlichieae | 34 |

**Table S2.**

**Synthesis of the numbers of subfamilies, tribes, genera and species in South America**

| **No** | **Subfamily** | **No tribes** | **No genera** | **No species** | **% tribes** | **% genera** | **% species** |
| --- | --- | --- | --- | --- | --- | --- | --- |
| 1 | Asteroideae | 16 | 390 | 5233 | 53.3 | 69.1 | 75.4 |
| 2 | Barnadesioideae | 1 | 10 | 95 | 3.3 | 1.8 | 1.4 |
| 3 | Carduoideae | 1 | 3 | 14 | 3.3 | 0.5 | 0.2 |
| 4 | Cichorioideae | 1 | 11 | 174 | 3.3 | 2.0 | 2.5 |
| 5 | Famatinanthoideae | 1 | 1 | 1 | 3.3 | 0.2 | 0.0 |
| 6 | Gochnatioideae | 1 | 5 | 52 | 3.3 | 0.9 | 0.7 |
| 7 | Mutisioideae | 3 | 42 | 440 | 10.0 | 7.4 | 6.3 |
| 8 | Stifftioideae | 1 | 10 | 44 | 3.3 | 1.8 | 0.6 |
| 9 | Vernonioideae | 3 | 86 | 850 | 10.0 | 15.2 | 12.2 |
| 10 | Wunderlichioideae | 2 | 6 | 37 | 6.7 | 1.1 | 0.5 |
|  | **Total** | **30** | **564** | **6940** | **100.0** | **100.0** | **100.0** |

**Table S3.**

**Representation of subfamilies in each country**

| **Subfamily** | **AR** | **BO** | **BR** | **CL** | **CO** | **EC** | **FG** | **GU** | **PA** | **PE** | **SU** | **UR** | **VE** |
| --- | --- | --- | --- | --- | --- | --- | --- | --- | --- | --- | --- | --- | --- |
| Asteroideae | 1 | 1 | 1 | 1 | 1 | 1 | 1 | 1 | 1 | 1 | 1 | 1 | 1 |
| Barnadesioideae | 1 | 1 | 1 | 1 | 1 | 1 | 0 | 0 | 1 | 1 | 0 | 1 | 1 |
| Carduoideae | 1 | 0 | 1 | 1 | 0 | 0 | 0 | 0 | 1 | 0 | 0 | 1 | 0 |
| Cichorioideae | 1 | 1 | 1 | 1 | 1 | 1 | 0 | 0 | 1 | 1 | 0 | 1 | 1 |
| Famatinanthoideae | 1 | 0 | 0 | 0 | 0 | 0 | 0 | 0 | 0 | 0 | 0 | 0 | 0 |
| Gochnatioideae | 1 | 1 | 1 | 1 | 0 | 1 | 0 | 0 | 1 | 1 | 0 | 1 | 1 |
| Mutisioideae | 1 | 1 | 1 | 1 | 1 | 1 | 0 | 1 | 1 | 1 | 1 | 1 | 1 |
| Stifftioideae | 1 | 1 | 1 | 0 | 1 | 0 | 1 | 1 | 0 | 0 | 0 | 0 | 1 |
| Vernonioideae | 1 | 1 | 1 | 0 | 1 | 1 | 1 | 1 | 1 | 1 | 1 | 1 | 1 |
| Wunderlichioideae | 1 | 1 | 1 | 0 | 1 | 1 | 0 | 1 | 1 | 1 | 0 | 1 | 1 |
| **Total subfamilies** | **10** | **8** | **9** | **6** | **7** | **7** | **3** | **5** | **8** | **7** | **3** | **8** | **8** |

**Table S4.**

**Number of species for each subfamily in each country**

| **Subfamily** | **AR** | **BO** | **BR** | **CL** | **CO** | **EC** | **FG** | **GU** | **PA** | **PE** | **SU** | **UR** | **VE** |
| --- | --- | --- | --- | --- | --- | --- | --- | --- | --- | --- | --- | --- | --- |
| Asteroideae | 982 | 910 | 1,447 | 558 | 1,072 | 748 | 52 | 104 | 375 | 1,273 | 52 | 258 | 634 |
| Barnadesioideae | 26 | 22 | 19 | 9 | 5 | 15 | 0 | 0 | 5 | 32 | 0 | 1 | 1 |
| Carduoideae | 1 | 0 | 1 | 14 | 0 | 0 | 0 | 0 | 1 | 0 | 0 | 1 | 0 |
| Cichorioideae | 76 | 39 | 13 | 54 | 22 | 25 | 0 | 0 | 8 | 32 | 0 | 12 | 8 |
| Famatinanthoideae | 1 | 0 | 0 | 0 | 0 | 0 | 0 | 0 | 0 | 0 | 0 | 0 | 0 |
| Gochnatioideae | 9 | 9 | 34 | 1 | 0 | 2 | 0 | 0 | 8 | 5 | 0 | 1 | 1 |
| Mutisioideae | 200 | 65 | 66 | 191 | 36 | 42 | 0 | 2 | 26 | 94 | 1 | 32 | 13 |
| Stifftioideae | 5 | 4 | 12 | 0 | 3 | 0 | 1 | 2 | 0 | 0 | 0 | 0 | 28 |
| Vernonioideae | 74 | 142 | 494 | 0 | 103 | 102 | 14 | 25 | 74 | 151 | 12 | 23 | 82 |
| Wunderlichioideae | 3 | 1 | 9 | 0 | 3 | 1 | 0 | 5 | 1 | 1 | 0 | 1 | 26 |
| **Total species** | 1,377 | 1,192 | 2,095 | 827 | 1,244 | 935 | 67 | 138 | 498 | 1,588 | 65 | 329 | 793 |

**Table S5.**

**Summary of species in IUCN categories**

| ID | Categ IUCN |  |
| --- | --- | --- |
| EX | Extinct | 1 |
| EW | Extinct in the Wild | 1 |
|  |  |  |
| CR | Critically Endangered * | 79 |
| EN | Endangered * | 159 |
| VU | Vulnerable * | 198 |
|  |  |  |
| NT | Near Threatened | 64 |
| LC | Least Concern | 229 |
| DD | Data Deficient | 46 |
|  |  |  |

* Categories CR, EN and VU are considered as Threatened (n=436)

**Table S6.**

**Species in IUCN categories by country**

| ID | Categ IUCN | AR | BO | BR | CL | CO | EC | FG | GU | PA | PE | SU | UR | VE |
| --- | --- | --- | --- | --- | --- | --- | --- | --- | --- | --- | --- | --- | --- | --- |
| 1 | Extinct | 0 | 0 | 0 | 0 | 0 | 1 | 0 | 0 | 0 | 0 | 0 | 0 | 0 |
| 2 | Extinct in the Wild | 1 | 0 | 0 | 0 | 0 | 0 | 0 | 0 | 0 | 0 | 0 | 0 | 0 |
| 3 | Critically Endangered | 0 | 0 | 8 | 14 | 26 | 25 | 0 | 0 | 0 | 1 | 0 | 0 | 6 |
| 4 | Endangered | 2 | 14 | 12 | 1 | 56 | 73 | 0 | 0 | 0 | 7 | 0 | 0 | 13 |
| 5 | Vulnerable | 0 | 14 | 6 | 2 | 37 | 140 | 0 | 0 | 0 | 10 | 0 | 0 | 11 |
| 6 | Near Threatened | 1 | 5 | 2 | 4 | 16 | 42 | 0 | 0 | 0 | 8 | 0 | 0 | 3 |
| 6 | Lower Risk/near threatened | 0 | 0 | 0 | 0 | 0 | 3 | 0 | 0 | 0 | 1 | 0 | 0 | 0 |
| 7 | Least Concern | 25 | 50 | 49 | 11 | 118 | 43 | 4 | 11 | 10 | 49 | 4 | 6 | 37 |
| 7 | Lower Risk/least concern | 0 | 0 | 0 | 0 | 0 | 2 | 0 | 0 | 0 | 1 | 0 | 0 | 0 |
| 8 | Data Deficient | 2 | 5 | 2 | 1 | 14 | 22 | 0 | 1 | 1 | 9 | 0 | 0 | 2 |
|  | **Total species evaluated** | **31** | **88** | **79** | **33** | **267** | **351** | **4** | **12** | **11** | **87** | **4** | **6** | **72** |
|  | **Total threatened species** | **2** | **28** | **26** | **17** | **119** | **238** | **0** | **0** | **0** | **18** | **0** | **0** | **30** |

**Table S7**

**Numbers of non-native or introduced species of Asteraceae in South America by each subfamily**

| **No** | **Subfamily** | **Tribes** | **Genera** | **Species** |
| --- | --- | --- | --- | --- |
| 1 | Asteroideae | 13 | 49 | 92 |
| 2 | Carduoideae | 2 | 11 | 31 |
| 3 | Vernonioideae | 2 | 4 | 5 |
| 4 | Cichorioideae | 1 | 21 | 47 |
| 5 | Mutisioideae | 1 | 1 | 2 |
|  | Total | 19 | 86 | 177 |

**Table S8**

**Numbers of non-native or introduced species of Asteraceae in South America by each tribe**

| **No** | **Subfamily** | **Tribe** | **Species** |
| --- | --- | --- | --- |
| 1 | Asteroideae | Anthemideae | 38 |
| 2 | Vernonioideae | Arctotideae | 4 |
| 3 | Asteroideae | Astereae | 8 |
| 4 | Asteroideae | Calenduleae | 4 |
| 5 | Carduoideae | Cardueae | 30 |
| 6 | Cichorioideae | Cichorieae | 47 |
| 7 | Asteroideae | Coreopsideae | 6 |
| 8 | Asteroideae | Eupatorieae | 3 |
| 9 | Asteroideae | Gnaphalieae | 3 |
| 10 | Asteroideae | Helenieae | 2 |
| 11 | Asteroideae | Heliantheae | 12 |
| 12 | Asteroideae | Inuleae | 1 |
| 13 | Asteroideae | Madieae | 1 |
| 14 | Asteroideae | Millerieae | 1 |
| 15 | Mutisioideae | Mutisieae | 2 |
| 16 | Asteroideae | Senecioneae | 12 |
| 17 | Asteroideae | Tageteae | 1 |
| 18 | Carduoideae | Tarchonantheae | 1 |
| 19 | Vernonioideae | Vernonieae | 1 |
|  |  | Total | 177 |

**Table S9. Most prolific taxonomists for the Asteraceae from South America**

| No | Descriptor | New species | Combination  (as basionym) | Combination  (as the person who  did the recombination) | Total |
| --- | --- | --- | --- | --- | --- |
|  | Example for Cuatrecasas | Cuatrec. | (Cuatrec.) B. Nord. | (Hook. & Arn.) Cuatrec. |  |
| 1 | Cuatrec. | 308 | 247 | 257 | 812 |
| 2 | R.M. King & H. Rob. | 156 | 1 | 551 | 708 |
| 3 | H. Rob. | 205 | 62 | 382 | 649 |
| 4 | Cabrera | 287 | 95 | 118 | 500 |
| 5 | DC. | 203 | 125 | 46 | 374 |
| 6 | Hieron. | 165 | 157 | 13 | 335 |
| 7 | B.L. Rob. | 122 | 173 | 20 | 315 |
| 8 | Baker | 122 | 91 | 50 | 263 |
| 9 | S.F. Blake | 150 | 66 | 44 | 260 |
| 10 | Kunth | 84 | 121 | 2 | 207 |

**Basic bibliography for the preparation of the South American Asteraceae Database:**

**Argentina**: Zuloaga, F. O., Morrone, O., and Belgrano, M. J. (ed.). (2008). Catálogo de las Plantas Vasculares del Cono Sur: Argentina, sur de Brasil, Chile, Paraguay y Uruguay. Vol 2. Monogr. Syst. Bot. Mo. Bot. Gard. 107; Zuloaga, F. O., Belgrano, M. J. and Anton, A. M. (ed.). (2014, 2015a, b). Flora Argentina: Flora Vascular de la República Argentina. Vol. 7(1, 2, 3). Buenos Aires: Estudio Sigma SRL; Flora del Cono Sur. (2021). San Isidro: Instituto de Botánica Darwinion. Available at <http://www.darwin.edu.ar/proyectos/floraargentina/fa.htm>

**Bolivia**: Hind, D. J. N. (2011). An annotated preliminary checklist of the Compositae of Bolivia 2. London: Royal Botanic Gardens Kew. Available at <https://www.kew.org/science/our-science/projects/annotatedsystematic-checklist-compositae-bolivia>; Jørgensen, P. M., Nee, M. H., and Beck, S. G. (ed.). 2014. Catálogo de las plantas vasculares de Bolivia. Monogr. Syst. Bot. Mo. Bot. Gard. 127.

**Brazil**: Roque, N., Nakajima, J., Heiden, G., Monge, M., Ritter, M. R., Loeuille, B. F. P., Christ, A. L., Rebouças, N. C., Castro, M. S., Teles, A. M., Saavedra, M. M., Gandara, A., Marques, D., Bringel Jr., J. B. A., Angulo, M. B., Souza-Buturi, F. O., Santos, J. U. M. D., Alves, M., Sancho, G., Reis-Silva, G. A., Volet, D. P., Hattori, E. K. O., Plos, A., Rivera, V. L., Carneiro, C. R., Simão-Bianchini, R., Magenta, M. A. G., Silva, G. H. L., Abreu, V. H. R., Bueno, V. R., Grossi, M. A., Amorim, V. O., Schneider, A. A., Borges, R. A. X., Siniscalchi, C. M., Via do Pico, G. M., Almeida, G. S. S., Freitas, F. S., Deble, L. P., Moreira, G. L., Contro, F. L., Gutiérrez, D. G., Souza-Souza, R. M. B., Viera Barreto, J. N., Picanço, W. L., Soares, P. N., Quaresma, A. S., Fernandes, F., Mondin, C. A., Salgado, V. G., Kilipper, J. T., Farco, G. E., Ribeiro, R. N., Walter, B. M. T., Lorencini, T. S., Fernandes, A. C., Silva, L. N., Barbosa, M. L., Semir, J., Barcelos, L. B., Ferreira, S. C., Dematteis, M., Moraes, M. D., Calvo, J., Bautista, H. P., and Hiriart, F. D. 2020. Asteraceae in Flora do Brasil 2020. Rio de Janeiro: Jardim Botânico do Rio de Janeiro. Available at <https://floradobrasil2020.jbrj.gov.br/FB55>; ; Flora del Cono Sur. (2021). San Isidro: Instituto de Botánica Darwinion. Available at <http://www.darwin.edu.ar/proyectos/floraargentina/fa.htm>

**Chile**: Zuloaga, F. O., Morrone, O., and Belgrano, M. J. (ed.). (2008). Catálogo de las Plantas Vasculares del Cono Sur: Argentina, sur de Brasil, Chile, Paraguay y Uruguay. Vol 2. Monogr. Syst. Bot. Mo. Bot. Gard. 107; Rodriguez, R., Marticorena, C., Alarcón, D., Baeza, C., Cavieres, L., Finot, V. L., Fuentes, N., Kiessling, A., Mihoc, M., Pauchard, A., Ruiz, E., Sanchez, P., and Marticorena, A. (2018). Catálogo de las plantas vasculares de Chile. Gayana Botánica 75, 1-430; Flora del Cono Sur. (2021). San Isidro: Instituto de Botánica Darwinion. Available at <http://www.darwin.edu.ar/proyectos/floraargentina/fa.htm>

**Colombia**: Bernal, R., Gradstein, S. R, and Celis, M. (ed.). (2019). Catálogo de plantas y líquenes de Colombia. Bogotá: Instituto de Ciencias Naturales, Universidad Nacional de Colombia. Available at <http://catalogoplantasdecolombia.unal.edu.co>

**Ecuador**: Jørgensen, P. M., and León-Yáñez, S. (ed.). (1999). Catalogue of the Vascular Plants of Ecuador. Monogr. Syst. Bot. Mo. Bot. Gard. 75.

**French Guiana**: Funk, V. A., Hollowell, T. H., Berry, P., Kelloff, C. L., and Alexander, S. N. (2007). Checklist of the plants of the Guiana Shield (Venezuela: Amazonas, Bolivar, Delta Amacuro; Guyana, Surinam, French Guiana). Contr. U.S. Natl. Herb. 55, 1-584.

**Guyana**: Funk, V. A., Hollowell, T. H., Berry, P., Kelloff, C. L., and Alexander, S. N. (2007). Checklist of the plants of the Guiana Shield (Venezuela: Amazonas, Bolivar, Delta Amacuro; Guyana, Surinam, French Guiana). Contr. U.S. Natl. Herb. 55, 1-584.

**Paraguay**: Zuloaga, F. O., Morrone, O., and Belgrano, M. J. (ed.). (2008). Catálogo de las Plantas Vasculares del Cono Sur: Argentina, sur de Brasil, Chile, Paraguay y Uruguay. Vol 2. Monogr. Syst. Bot. Mo. Bot. Gard. 107; Flora del Cono Sur. (2021). San Isidro: Instituto de Botánica Darwinion. Available at <http://www.darwin.edu.ar/proyectos/floraargentina/fa.htm>

**Peru**: Brako, L., and Zarucchi, J. L. (ed.). (1993). Catalogue of the Flowering Plants and Gymnosperms of Peru. Monogr. Syst. Bot. Mo. Bot. Gard. 45; Beltrán, H. (2019). Nuevos registros de asteráceas para la flora del Perú. Arnaldoa 26, 839-448.

**Suriname**: Funk, V. A., Hollowell, T. H., Berry, P., Kelloff, C. L., and Alexander, S. N. (2007). Checklist of the plants of the Guiana Shield (Venezuela: Amazonas, Bolivar, Delta Amacuro; Guyana, Surinam, French Guiana). Contr. U.S. Natl. Herb. 55, 1-584.

**Uruguay**: Zuloaga, F. O., Morrone, O., and Belgrano, M. J. (ed.). (2008). Catálogo de las Plantas Vasculares del Cono Sur: Argentina, sur de Brasil, Chile, Paraguay y Uruguay. Vol 2. Monogr. Syst. Bot. Mo. Bot. Gard. 107; Flora del Cono Sur. (2021). San Isidro: Instituto de Botánica Darwinion. Available at <http://www.darwin.edu.ar/proyectos/floraargentina/fa.htm>

**Venezuela**: Badillo, V. M. (2001). Lista actualizada de las especies de la familia Compuestas (Asteraceae) de Venezuela. Ernstia 11, 147-215; Funk, V. A., Hollowell, T. H., Berry, P., Kelloff, C. L., and Alexander, S. N. (2007). Checklist of the plants of the Guiana Shield (Venezuela: Amazonas, Bolivar, Delta Amacuro; Guyana, Surinam, French Guiana). Contr. U.S. Natl. Herb. 55, 1-584.

This Supplementary Material includes a Dataset S1 in Excel format: **Catalogue of the Asteraceae of South America.**
